# Significant Modules and Biological Processes between Active Components of* Salvia miltiorrhiza* Depside Salt and Aspirin

**DOI:** 10.1155/2016/3439521

**Published:** 2016-03-16

**Authors:** Yuan Li, Yanming Xie, Lianxin Wang, Yingying Zhang, Hao Gu, Yan Chai

**Affiliations:** ^1^Institute of Basic Research in Clinical Medicine, China Academy of Chinese Medical Sciences, No. 16 Nanxiao Street, Dongzhimennei, Dongcheng District, Beijing 100700, China; ^2^Department of Epidemiology, University of California, Los Angeles, 405 Hilgard Avenue, CA 90095, USA

## Abstract

The aim of this study is to examine and compare the similarities and differences between active components of* S. miltiorrhiza* depside salt and aspirin using perspective of pharmacological molecular networks. Active components of* S. miltiorrhiza* depside salt and aspirin's related genes were identified via the STITCH4.0 and GeneCards Database. A text search engine (Agilent Literature Search 2.71) and MCODE software were applied to construct network and divide modules, respectively. Finally, 32, 2, and 28 overlapping genes, modules, and pathways were identified between active components of* S. miltiorrhiza* depside salt and aspirin. A multidimensional framework of drug network showed that two networks reflected commonly in human aortic endothelial cells and atherosclerosis process. Aspirin plays a more important role in metabolism, such as the well-known AA metabolism pathway and other lipid or carbohydrate metabolism pathways.* S. miltiorrhiza* depside salt still plays a regulatory role in type II diabetes mellitus, insulin resistance, and adipocytokine signaling pathway. Therefore, this study suggests that aspirin combined with* S. miltiorrhiza* depside salt may be more efficient in treatment of CHD patients, especially those with diabetes mellitus or hyperlipidemia. Further clinical trials to confirm this hypothesis are still needed.

## 1. Introduction 

Antithrombotic Therapy and Prevention of Thrombosis (Version 9) [1], announced by the American College of Chest Physicians (ACCP) Evidence-Based Clinical Practice Guidelines, proposed lasting low-dose aspirin therapy to be used as the primary prevention technique for patients above 50 years old or patients diagnosed with coronary heart disease (CHD). For patients with acute coronary syndrome (ACS) or undergoing stent implantation with PCI, dual antiplatelet therapy for up to one year is required. Antithrombotic therapy plays a crucial role in prevention and treatment of CHD, in which aspirin undoubtedly is the most widely used conventional drug. Aspirin irreversibly inhibits COX-1 and modifies the enzymatic activity of COX-2, which normally produces prostanoids [2]. Antiplatelet effect of aspirin inhibits the prostaglandin production which downregulates thromboxane A2 (TXA2) levels. TXA2 is bound by platelet molecules under the normal circumstances to create a patch over damaged walls of blood vessels. Due to the fact that it inhibits formation of blood clot in people with high risk [3], aspirin is also used in the long term, at low doses, to help prevent heart attacks [4].* Salvia miltiorrhiza*, a Chinese herbal medicine which promotes blood circulation to remove blood stasis drugs [5], has been widely used to treat cardiovascular diseases such as CHD. In 2005,* S. miltiorrhiza* depside salt passed the certification of new drug application for chronic angina treatment at the State Food and Drug Administration (SFDA). 80% of its active components are magnesium lithospermate B (MLB) and its analogs (salvianolic acid B and lithospermic acid B) [6] extracted from* Salvia miltiorrhiza* major water-soluble active ingredients. The other 20% are mainly rosmarinic acid (RA) and lithospermic acid (LA). A clinical noninferiority study showed that* S. miltiorrhiza* depside salt had definite therapeutic effect in patients with CHD angina pectoris, with no evidence of adverse drug reaction (ADR) [7]. Aspirin and* S. miltiorrhiza* depside salt are thus commonly used in CHD treatment, but the molecular relationships between the two drugs are still under study. Current evidence shows that both similar and different molecular therapeutic patterns exist between these two drugs, but the exact pattern of whether they are the same, different, or partially overlapping remains unknown. Therefore, a network pharmacology approach seems to be of interest as it would reveal the potential overlapping or unique modules that are affected by either treatment.

“Network pharmacology” [8], which combines systems biology and biological networks, balances the perspective of development process to explain the disease [9]. It improves or restores biological networks from a balanced perspective and explains the interaction between the drug and the human body. It emphasizes the discovery of drugs' signaling pathway and provides a reference to improve the therapeutic effect and reduce side effects. According to the characteristics of the multicomponent and multitarget of Chinese herbal medicine, network pharmacology may be a new and well-documented method to find some meaningful information. Therefore, related genes were used to construct the molecular network, combined with module division with modular analysis to excavate the associations of aspirin and active components of S*. miltiorrhiza* depside salt in this study. We believe that exploring the internal connection of potential molecular interactions between the two drugs can provide a clue for combination therapy of CHD.

## 2. Materials and Methods

### 2.1. Gene Obtaining

GeneCards (http://www.genecards.org/) is a comprehensive and authoritative database that provides information of human genes [10] and is known as the most inclusive resource of gene-centered information of the human genome [11]. STITCH (http://stitch.embl.de/) is a database of protein-chemical interactions integrating massive literature information and various databases of biological pathways and drug-target relationships [12]. It is used to create a network of interactions [13]. “MLB”, “RA”, “LA” (active components of* S. miltiorrhiza* depside salt), and “Aspirin” were entered into the integrated and searchable database of GeneCards and STITCH4.0 to search and export related genes in* Homo sapiens*, respectively.

### 2.2. Network Construction

The genes related to active components of* S. miltiorrhiza* depside salt (“MLB”, “RA”, and “LA”) and “Aspirin” were submitted to the Agilent Literature Search software v.3.1.1 (http://www.agilent.com/labs/research/litsearch.html), which is a powerful automatic metasearch tool for querying multiple text-based PubMed and USPTO, for associations among genes of interest and constructing a network. Agilent Literature Search is a registered plugin and can be used in conjunction with Cytoscape (http://www.cytoscape.org/) to realize the visualization and analyzation of the network [14] (parameters: Max Engine Matches = 10; Concept Lexicon =* Homo sapiens*; Interaction Lexicon = limited).

### 2.3. Identification of Modules

The modular structures exist in a complex biological systematic network, so we detect highly interconnected regions clusters in the network by MCODE (http://baderlab.org/Software/MCODE). MCODE is a clustering algorithm tool, which divides network module and provides detailed algorithm [15] (parameters: degree cutoff = 2; K-core = 2; node score cutoff = 0.2). Network can be divided into several modules. Results can be visualized by Cytoscape.

### 2.4. GO Biological Process and KEGG Pathway Enrichment

DAVID software was used (http://david.abcc.ncifcrf.gov/) to analyze the function of modules. DAVID software provides hypergeometric distribution tests and mainly includes typical batch annotation and gene-GO term enrichment analysis to highlight the most relevant GO terms associated with a given gene list [16] (parameters: Count = 2; EASE = 0.01; species and background =* Homo sapiens*). DAVID functional annotation clustering uses an algorithm to measure relationships among the annotation terms based on the degrees of their coassociation genes to group similar, redundant, and heterogeneous annotation contents from the same or different resources into annotation groups. It can display genes from a user's list on pathway maps to facilitate biological interpretation in a network context by taking full advantage of the well-known KEGG pathways. The biological processes and KEGG pathway corresponding to the modules were identified and ranked by *P* values (*P* < 0.05; reliability is higher).

## 3. Result

### 3.1. Genes Related to Active Components of* S. miltiorrhiza* Depside Salt and Aspirin in STITCH and GeneCards Database

After searching the STITCH (*Homo sapiens*, score > 0.400, medium confidence) and GeneCards Database (on March 16, 2015), we found 55 genes related to active components of* S. miltiorrhiza* depside salt and 498 genes related to aspirin. There were 32 overlapping genes shared by the two drugs, and these genes accounted for 58.18% (32/55) of the identified genes related to active components of* S. miltiorrhiza* depside salt, 6.43% (32/498) of the identified genes related to aspirin, and 5.79% (32/553) of all genes related to both drugs. After entering 32 overlapping genes' list in GO functional annotation, it showed that the common roles included response to chemical stimulus, response to stress, multiorganism process, response to stimulus, and response to external stimulus (Table 1).

### 3.2. Topological Analysis of Network

**Figure 1:**
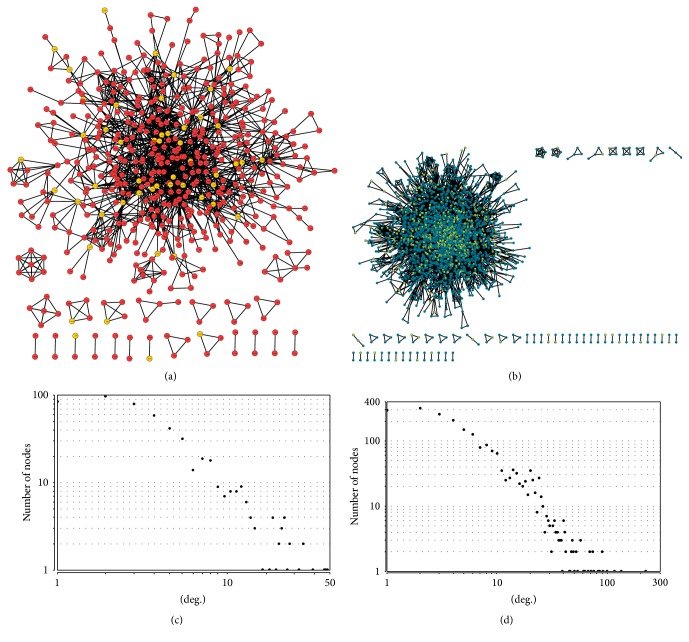
Characteristics of two gene interaction networks. (a) Active components of* S. miltiorrhiza* depside salt-related genes interaction network. (b) Aspirin-related genes interaction network. Yellow diamonds denote known active components of* S. miltiorrhiza* depside salt and aspirin-related genes, and red and green denote genes obtained from text mining. (c) The degree distribution of active components of* S. miltiorrhiza* depside salt network. (d) The degree distribution of aspirin network.

After submitting 55 and 498 genes related to active components of* S. miltiorrhiza* depside salt and aspirin into the Agilent Literature Search 3.1.1, two networks were created (Figures 1(a) and 1(b)). There were 528 nodes (genes) and 1506 edges (interactions) identified from the active components of* S. miltiorrhiza* depside salt-related genes and 2120 nodes (genes) and 9064 edges (interactions) from the aspirin-related genes. Similar distributions of node degree that followed the power-law distribution appeared in two networks (Figures 1(c) and 1(d)). The topological analysis of two networks such as clustering coefficient, centralization, density, diameter, and radius was shown in Table 2.

### 3.3. Module Identification

**Figure 2:**
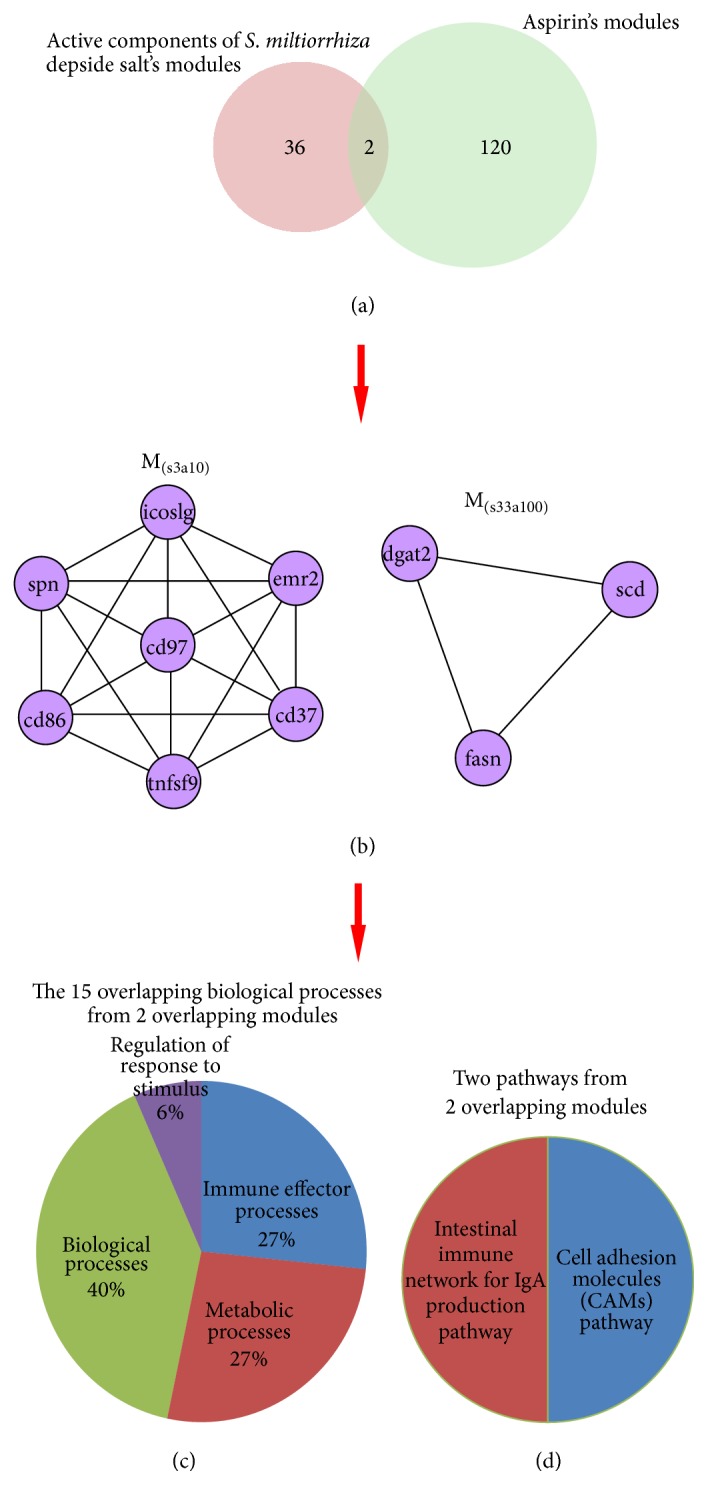
Number of two-drug modules, overlapping modules, and biological functions. (a) Number of modules identified from two networks. (b) Blue circles indicate the genes in the 2 overlapping modules. (c) GO biological processes of 2 overlapping modules. (d) Pathways of 2 overlapping modules.

After submitting two networks into MCODE software, 38 modules were identified from active components of* S. miltiorrhiza* depside salt network. The maximum module was composed of 46 nodes while the minimum was composed of 3 nodes. There were 122 modules in aspirin network. The maximum module is composed of 112 nodes while the minimum is composed of 3 nodes. Two networks shared 2 overlapping functional modules including the same genes: M_(s3a10)_ and M_(s33a100)_ (Figure 2(a)). M_(s3a10)_ contains 7 nodes (CD86, TNFSF9, EMR2, CD37, ICOSLG, SPN, and CD97) and 21 edges. M_(s33a100)_ contains 3 nodes (FASN, SCD, and DGAT2) and 3 edges (Figure 2(b)).

### 3.4. Functional Enrichment Analysis of 2 Overlapping Modules

The two overlapping modules contained 15 biological functional annotations and 2 pathways (Figure 2(c)). Biological annotations included immune effector processes (4) (regulation of immune response, regulation of immune effector process, positive regulation of immune system process, and regulation of immune system process), lipid biosynthetic and metabolic processes (5) (lipid biosynthetic process, lipid metabolic processes, fatty acid metabolic process, cellular lipid metabolic process, and fatty acid metabolic process), other biosynthetic and metabolic processes (5) (carboxylic acid biosynthetic process, organic acid biosynthetic process, cellular biosynthetic process, monocarboxylic acid metabolic process, and biosynthetic process), and regulation of response to stimulus (1). So the main biological functions of the 2 overlapping modules were lipid biosynthetic and metabolic and immune effector. The 2 pathways from overlapping modules were cell adhesion molecules (CAMs) pathway and intestinal immune network for IgA production pathway.

### 3.5. Functional Enrichment of Unique Modules for 2 Drugs

**Figure 3:**
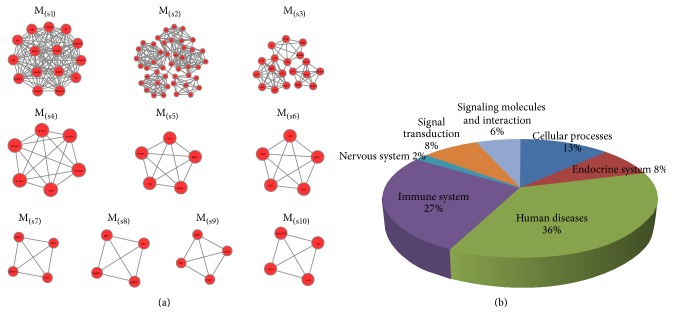
Top 10 unique modules and their pathways of active components of* S. miltiorrhiza* depside salt. (a) The top 10 unique modules for active components of* S. miltiorrhiza* depside salt network. (b) The 63 KEGG pathways identified from top 10 unique modules that divided into 7 categories.

**Figure 4:**
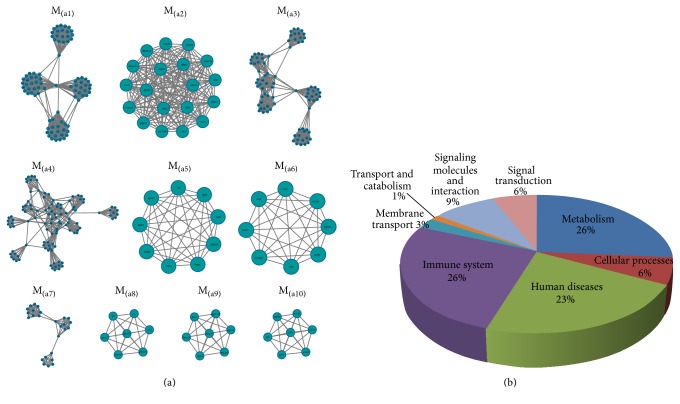
Top 10 unique modules and their pathways of aspirin. (a) The top 10 unique module networks for aspirin network. (b) The 80 KEGG pathways identified from top 10 unique modules that divided into 9 categories.

GO functional enrichment analysis was implemented on acquired top 10 nonoverlapping modules of the 2 drugs sorted by MCODE score, respectively (Figures 3(a) and 4(a)). 762 GO biological functions and 63 KEGG pathways were found in modules of active components of* S. miltiorrhiza* depside salt's network, and 1391 GO biological functions and 80 KEGG pathways were found in modules of aspirin's network. The 63 pathways of Salvianolate network (Figure 3(b)) are as follows: 23 human diseases pathways (cancers (pancreatic cancer, bladder cancer, and another 17 cancers), immune diseases (primary immunodeficiency and autoimmune thyroid disease), endocrine and metabolic diseases (type II diabetes mellitus), and infectious diseases (epithelial cell signaling in* Helicobacter pylori* infection)), 17 immune system pathways, 8 cellular processes pathways (cell growth and death (6), regulation of autophagy, and focal adhesion), 5 endocrine system pathways, and so forth. The 80 pathways (Figure 4(b)) of aspirin network are as follows: 21 metabolism pathways (carbohydrate metabolism (6), xenobiotics biodegradation (5), cofactors and vitamins metabolism (3), lipid metabolism (3), and others), 21 immune system pathways (hematopoietic cell lineage, natural killer cell mediated cytotoxicity, Toll-like receptor signaling pathway, etc.), 18 human diseases pathways (cancers (prostate cancer, prostate cancer, and another 9 cancers), immune diseases (5), viral myocarditis (1), and Alzheimer's disease (1)), 7 signaling molecules and interaction pathways, 5 cellular processes (cell growth and death (3) and cellular community (2)), 5 signal transduction pathways, and so forth.

After eliminating repeated pathways, active components of* S. miltiorrhiza* depside salt had 44 pathways while aspirin had 53 pathways, including 26 common pathways. Common pathways were as follows: immune systems (11) (chemokine signaling pathway, NOD-like receptor signaling pathway, hematopoietic cell lineage pathway, T cell receptor signaling pathway, Toll-like receptor signaling pathway, RIG-I-like receptor signaling pathway, and 5 other related immune system pathways), human diseases (5) (cancers (bladder cancer, small cell lung cancer, and colorectal cancer and pathways in cancer) autoimmune thyroid disease), cellular processes (3) (apoptosis, p53 signaling pathway, and focal adhesion), signal transduction (3) (ErbB signaling pathway, Jak-STAT signaling pathway, and VEGF signaling pathway), signaling molecules and interactions (2) (CAMs and cytokine-cytokine receptor interaction), neurotrophin signaling pathway (1), and progesterone-mediated oocyte maturation. Unique pathways of active components of* S. miltiorrhiza* depside salt were as follows: cancers (6), immune diseases or systems (4) (autoimmune thyroid disease, primary immunodeficiency, B cell receptor signaling pathway, and Fc gamma R-mediated phagocytosis pathway), endocrine diseases or systems (4) (insulin signaling pathway, type II diabetes mellitus, adipocytokine signaling pathway, GnRH signaling pathway, and progesterone-mediated oocyte maturation), cellular processes (3) (cell cycle, regulation of autophagy, and oocyte meiosis), and mTOR signaling pathway (1). Unique pathways of aspirin were as follows: metabolism pathways (17) (arachidonic acid metabolism, linoleic acid metabolism, other carbohydrate metabolisms, and another 14 related pathways), immune diseases and system (4) (Huntington's disease, allograft rejection, amyotrophic lateral sclerosis (ALS) pathway, and complement and coagulation cascades pathway), human diseases (3) (viral myocarditis, Alzheimer's disease, and Parkinson's disease), PPAR signaling pathway (1), ABC transporters pathway (1), and ECM-receptor interaction pathway (1).

## 4. Discussion

Aspirin and* S. miltiorrhiza* depside salt are commonly used drugs for the treatment of CHD, but there is lack of relationship studies between the two drugs from large-scale molecular perspective. Two overlapping modules and many common biological processes and pathways between the two drugs were known in this investigation. These results are likely to reveal the overlap of potential therapy mechanism and internal connection between the two drugs. Except for the overlap, the two drugs also have some unique functions, which have an excellent advantage in different ranges.

There are 32 overlapping genes between aspirin and active components of* S. miltiorrhiza* depside salt, such as JUN, VCAM-1, TGFB1, TGFB1, IL8, IL1B, NOS2, NOS3, MAPK1, MAPK8, CASP3, MMP1, and MMP2 that have been associated with the cardiovascular diseases (coronary heart disease, angina pectoris, atherosclerosis, and thrombosis which are all within the scope of CHD) as biomarkers or therapeutic targets from the Comparative Toxicogenomics Database. Vascular endothelial cell proliferation and apoptosis are an early marker for atherosclerosis. Therefore, prevention of smooth muscle cell and endothelial cell proliferation and removal of superoxide radicals have a positive effect for treatment of CHD. JUN participates in the biological process of angiogenesis [17] and increases endothelial cell proliferation and smooth muscle cell hyperplasia [18]. IL8, IL1B, TGFB1, and VCAM-1 participate in the inflammatory response [19]. TGFB1 positively regulates cell migration, apoptosis, and blood vessel endothelial cell migration [20]. VCAM-1, which belongs to immunoglobulin superfamily (IgSF), is widely expressed in human aortic endothelial cells (HAECs). It also plays an important role in cell adhesion. Overexpression of VCAM-1 causes increased endothelial adhesion [21], which further leads to the formation of atherosclerotic plaque rupture NOS2 and NOS3 belonging to NO (produces nitric oxide) [22], which is implicated in vascular smooth muscle relaxation through a cGMP-mediated signal transduction pathway. NOS2 and NOS3 positively regulate vasodilation and mediate vascular endothelial growth factor- (VEGF-) induced angiogenesis in coronary vessels [23] and promote blood coagulation through the activation of platelets [24]. MAPK1, MAPK8, and CASP3 play an irreplaceable role in biological process of apoptosis. MAPK is a key signal transduction receptor from the surface to the nucleus, which is involved in cell proliferation, differentiation, migration, transformation, and apoptosis in the whole process. MAPKs play an important role in regulating the expression of proinflammatory molecules in many cells [25]. CASP3 can not only activate a variety of factors for apoptosis [26], but also participate in the process of platelet formation [27]. MMP1 and MMP2 belong to the metalloproteinase family. They are involved in diverse functions such as remodeling of the vasculature, angiogenesis, inflammation, blood coagulation, and atherosclerotic plaque rupture. MMPs have a role in myocardial cell death pathways and vascular remodeling [28], such as vascular smooth muscle cell migration into the intima [29]. Regulation of cell proliferation, apoptosis, programmed cell death, and cell death are common biological functions of the 32 overlapping genes, which are considered remarkable aspects of CHD [30]. Thus, these overlapping genes' biological functions are mainly reflected in HAECs and atherosclerosis process.

There are 28 common pathways in overlapping and top 10 unique modules between active components of* S. miltiorrhiza* depside salt and aspirin. Most of these pathways are related to the antitumor and inflammatory immune response (p53 signaling pathway, ErbB signaling pathway, T cell receptor signaling pathway, etc.). But there are also some pathways participating in the process of antiatherosclerosis. CAMs pathway participates in cell growth and death, which are associated with cardiovascular diseases [31]. CAMs are proteins expressed on the cell surface and function in HAECs adhesion. CAMs play a critical role in a wide array of biological processes that include hemostasis, immune response, inflammation, embryogenesis, and development of neuronal tissue, which establish strong adhesion on the endothelium of arteries. VEGF signaling pathway can regulate vascular endothelial cell proliferation and migration. It also leads to change of vascular permeability and control of angiogenesis. There is now much evidence that VEGFR-2 is the major mediator of VEGF-driven responses in endothelial cells and it is considered to be a crucial signal transducer in both physiologic and pathologic angiogenesis [32]. JAK-STAT pathway is the principal signaling mechanism for a wide array of cytokines and growth factors, closely related to the main factor VEGF which mediates proliferation and migration of vascular endothelial cells [33]. In IFN-*γ*-treated HAECs, MLB inhibited IFN-*γ*-induced JAK-STAT signaling pathways and consequently suppressed IFN-*γ*-induced expression of chemokines, IP-10 promoter activity, IP-10 protein release, and monocyte adhesion to HAECs [34].

Antiplatelet treatment with aspirin is widely considered as a cornerstone of atherosclerotic vascular disease's primary [35] and secondary prevention and also used for acute treatment [36]. In this study, we found that aspirin may play a more important role in metabolism, such as the well-known AA metabolism pathway and other lipid or carbohydrate metabolism pathways. Moreover, we found that aspirin participates in the PPAR signaling pathway, and PPAR-*γ* has played a pivotal role in anti-inflammation, atherosclerosis, insulin resistance, and antitumor [37]. Active components of* S. miltiorrhiza* depside salt may play a more important role in endocrine system, such as type II diabetes mellitus, insulin signaling pathway, and adipocytokine signaling pathway. Cardiovascular disease, especially CHD, is the primary cause of mortality among diabetes mellitus patients. Entirely due to more extensive coronary atherosclerosis, more than 50% of diabetes mellitus patients will die from a cardiovascular event. Meanwhile, diabetes mellitus is a risk factor for coronary heart disease [38]. Type II diabetes mellitus is a chronic low-grade inflammatory disease. Inflammatory factor and adipose cytokines are involved in the development of type II diabetes mellitus and its complications [39]. The metabolic syndrome can promote the development of type II diabetes and CHD. Insulin resistance plays a pivotal role in the progression of this syndrome and cardiovascular diseases. Improvement of insulin resistance, therefore, is most likely to reduce the high cardiovascular event rate in type II diabetes [40]. Control of blood glucose and blood lipid has a positive role to prevent and delay the development of atherosclerosis. Previous studies have demonstrated that Salvianolate has positive effect on attenuating atherosclerosis [41], scavenging free radical [42], preventing endothelial dysfunction [43], regulating matrix metalloproteinases expression, activity, and anti-inflammation [44], modulating lipid profiles [45], and protecting against myocardial ischemia and reperfusion (MI/R) injury [46].

Finally, through analyzing the molecular networks module, these two drugs were found not only sharing certain biological processes and pathways, but also having unique character. So using aspirin combined with* S. miltiorrhiza* depside salt may be more efficient in treatment of CHD patients, especially those with diabetes mellitus or hyperlipidemia.

## 5. Conclusion

We adopted network-based approach to investigate the similarities and differences between* S. miltiorrhiza* depside salt and aspirin in great detail. A multidimensional framework of drug network showed that the two drugs reflected commonly in human aortic endothelial cells and atherosclerosis process. Aspirin plays a more important role in metabolism, such as the well-known AA metabolism pathway and other lipid or carbohydrate metabolism pathways.* S. miltiorrhiza* depside salt still plays a regulatory role in type II diabetes mellitus, insulin resistance, and adipocytokine signaling pathway. Therefore, this study suggests that aspirin combined with* S. miltiorrhiza* depside salt may be more efficient in treatment of CHD patients, especially those with diabetes mellitus or hyperlipidemia, but should be confirmed by further clinical trials.

## Supplementary Material

There are 5 tables in the Supplementary Material file. Table 1 contained the detailed genes related to active components of S. miltiorrhiza depside salt and aspirin. Table 2 and table 3 contained the modules of active components of S. miltiorrhiza depside salt and aspirin network, and ever modules' related genes. Table 4 and table 5 contained the detail of every biological function and KEGG pathway related to active components of S. miltiorrhiza and aspirin.

## Figures and Tables

**Table 1:** Top 10 overlapping biological functions of the 32 overlapping genes related to active components of *S. miltiorrhiza* depside salt and aspirin.

GO terms	*P* value	Gene
GO:0042221: response to chemical stimulus	9.5*E* − 19	XDH, TNF, MCL1, IL8, PTGS2, RELA, EDN1, SOD1, MMP2, TGFB1, CCL11, MAPK1, CASP3, CCR3, JUN, BCL2, SERPINE1, IFNG, IL1B, NOS3, MAPK8, ALOX5, NOS2, and IKBKB

GO:0006950: response to stress	4.3*E* − 16	TNF, PTGER3, IL8, PTGS2, RELA, EDN1, NFKB1, SOD1, MMP2, TGFB1, CCL11, MAPK1, CASP3, CCR3, JUN, BCL2, AKR1B1, SERPINE1, IFNG, IL1B, NOS3, MAPK8, ALOX5, and NOS2

GO:0051704: multiorganism process	5.6*E* − 14	TNF, PTGS2, RELA, SOD1, MMP1, TGFB1, CCL11, VCAM1, MAPK1, APP, CCR3, JUN, BCL2, IFNG, IL1B, NOS3, and NOS2

GO:0050896: response to stimulus	7.5*E* − 14	XDH, TNF, MCL1, PTGS2, EDN1, NFKB1, MMP2, TGFB1, CASP3, APP, BCL2, SERPINE1, IFNG, IL1B, NOS3, NOS2, PTGER3, IL8, RELA, SOD1, CCL11, MAPK1, CCR3, JUN, AKR1B1, MAPK8, ALOX5, and IKBKB

GO:0009605: response to external stimulus	2.9*E* − 13	TNF, PTGER3, IL8, PTGS2, RELA, NFKB1, SOD1, TGFB1, CCL11, MAPK1, CASP3, CCR3, JUN, BCL2, IFNG, SERPINE1, IL1B, and ALOX5

GO:0042127: regulation of cell proliferation	5.2*E* − 13	PTGER2, TNF, IL8, PTGS2, RELA, EDN1, TGFB1, VCAM1, MAPK1, CASP3, JUN, BCL2, IFNG, SERPINE1, IL1B, NOS3, and NOS2

GO:0042981: regulation of apoptosis	7.3*E* − 13	TNF, PTGS2, MCL1, RELA, NFKB1, SOD1, TGFB1, MAPK1, APP, CASP3, JUN, BCL2, IFNG, IL1B, NOS3, MAPK8, and IKBKB

GO:0043067: regulation of programmed cell death	8.5*E* − 13	TNF, PTGS2, MCL1, RELA, NFKB1, SOD1, TGFB1, MAPK1, APP, CASP3, JUN, BCL2, IFNG, IL1B, NOS3, MAPK8, and IKBKB

GO:0010941: regulation of cell death	9*E* − 13	TNF, PTGS2, MCL1, RELA, NFKB1, SOD1, TGFB1, MAPK1, APP, CASP3, JUN, BCL2, IFNG, IL1B, NOS3, MAPK8, and IKBKB

GO:0065008: regulation of biological quality	3.4*E* − 12	XDH, TNF, PTGER3, PTGS2, MCL1, EDN1, SOD1, TGFB1, CCL11, CASP3, APP, CCR3, JUN, BCL2, IFNG, SERPINE1, IL1B, NOS3, NOS2, and IKBKB

**Table 2:** The topological attributes of the gene interaction networks.

Parameters	Active components of *S. miltiorrhiza* depside salt	Aspirin
Clustering coefficient	0.618	0.586
Nodes	528	2120
Edges	1506	9064
Network centralization	0.081	0.101
Network density	0.011	0.004
Network diameter	10	9
Network radius	1	1

## References

[1] Holbrook A., Schulman S., Witt D. M. (2012). Evidence-based management of anticoagulant therapy: antithrombotic therapy and prevention of thrombosis, 9th ed: American College of Chest Physicians Evidence-Based Clinical Practice Guidelines. *Chest*.

[2] Morita I. (2002). Distinct functions of COX-1 and COX-2. *Prostaglandins and Other Lipid Mediators*.

[3] Lewis H. D., Davis J. W., Archibald D. G. (1983). Protective effects of aspirin against acute myocardial infarction and death in mean with unstable angina. Results of a veterans administration cooperative study. *New England Journal of Medicine*.

[4] Julian D. G., Chamberlain D. A., Pocock S. J. (1996). A comparison of aspirin and anticoagulation following thrombolysis for myocardial infarction (the AFTER study): a multicentre unblinded randomised clinical trial. *British Medical Journal*.

[5] Zhou L., Zuo Z., Chow M. S. S. (2005). Danshen: an overview of its chemistry, pharmacology, pharmacokinetics, and clinical use. *Journal of Clinical Pharmacology*.

[6] Watzke A., O'Malley S. J., Bergman R. G., Ellman J. A. (2006). Reassignment of the configuration of salvianolic acid B and establishment of its identity with lithospermic acid B. *Journal of Natural Products*.

[7] Zhang Q., Liu A.-D., Huang Y.-S. (2006). Clinical non-inferiority trial on treatment of coronary heart disease angina pectoris of Xin-blood stasis syndrome type with lyophilized Salvia salt of lithospermic acid powder for injection. *Chinese Journal of Integrative Medicine*.

[8] Hopkins A. L. (2007). Network pharmacology. *Nature Biotechnology*.

[9] Hopkins A. L. (2008). Network pharmacology: the next paradigm in drug discovery. *Nature Chemical Biology*.

[10] Safran M., Dalah I., Alexander J. (2010). GeneCards Version 3: the human gene integrator. *Database*.

[11] Shklar M., Strichman-Almashanu L., Shmueli O., Shmoish M., Safran M., Lancet D. (2005). GeneTide—Terra Incognita Discovery Endeavor: a new transcriptome focused member of the GeneCards/GeneNote suite of databases. *Nucleic Acids Research*.

[12] Kuhn M., Szklarczyk D., Franceschini A. (2009). STITCH 2: an interaction network database for small molecules and proteins. *Nucleic Acids Research*.

[13] Kuhn M., Szklarczyk D., Pletscher-Frankild S. (2014). STITCH 4: integration of protein-chemical interactions with user data. *Nucleic Acids Research*.

[14] Su G., Morris J. H., Demchak B., Bader G. D. (2014). Biological network exploration with cytoscape 3. *Current Protocols in Bioinformatics*.

[15] Rivera C. G., Vakil R., Bader J. S. (2010). NeMo: network module identification in cytoscape. *BMC Bioinformatics*.

[16] Burkard T. R., Rix U., Breitwieser F. P., Superti-Furga G., Colinge J. (2010). A computational approach to analyze the mechanism of action of the kinase inhibitor bafetinib. *PLoS Computational Biology*.

[17] Folkman J. (2004). Angiogenesis and c-Jun. *Journal of the National Cancer Institute*.

[18] Pedram A., Razandi M., Levin E. R. (1998). Extracellular signal-regulated protein kinase/jun kinase cross-talk underlies vascular endothelial cell growth factor-induced endothelial cell proliferation. *The Journal of Biological Chemistry*.

[19] Tille J.-C., Pepper M. S. (2002). Mesenchymal cells potentiate vascular endothelial growth factor-induced angiogenesis in vitro. *Experimental Cell Research*.

[20] Benckert C., Jonas S., Cramer T. (2003). Transforming growth factor *β*1 stimulates vascular endothelial growth factor gene transcription in human cholangiocellular carcinoma cells. *Cancer Research*.

[21] Galkina E., Ley K. (2007). Vascular adhesion molecules in atherosclerosis. *Arteriosclerosis, Thrombosis, and Vascular Biology*.

[22] O'Brien K. D., McDonald T. O., Chait A., Allen M. D., Alpers C. E. (1996). Neovascular expression of E-selectin, intercellular adhesion molecule-1, and vascular cell adhesion molecule-1 in human atherosclerosis and their relation to intimal leukocyte content. *Circulation*.

[23] Capettini L. S. A., Cortes S. F., Silva J. F., Alvarez-Leite J. I., Lemos V. S. (2011). Decreased production of neuronal NOS-derived hydrogen peroxide contributes to endothelial dysfunction in atherosclerosis. *British Journal of Pharmacology*.

[24] Li H., Förstermann U. (2009). Prevention of atherosclerosis by interference with the vascular nitric oxide system. *Current Pharmaceutical Design*.

[25] Zhang S., Yuan J., Yu M. (2012). IL-17A facilitates platelet function through the ERK2 signaling pathway in patients with acute coronary syndrome. *PLoS ONE*.

[26] Nassef Y. E., Hamed M. A., Aly H. F. (2014). Inflammatory cytokines, apoptotic, tissue injury and remodeling biomarkers in children with congenital heart disease. *Indian Journal of Clinical Biochemistry*.

[27] Wang M., Sun G.-B., Sun X. (2013). Cardioprotective effect of salvianolic acid B against arsenic trioxide-induced injury in cardiac H9c2 cells via the PI3K/Akt signal pathway. *Toxicology Letters*.

[28] Fan W.-H., Karnovsky M. J. (2002). Increased MMP-2 expression in connective tissue growth factor over-expression vascular smooth muscle cells. *The Journal of Biological Chemistry*.

[29] Sweeney N. V. O., Cummins P. M., Birney Y. A., Redmond E. M., Cahill P. A. (2004). Cyclic strain-induced endothelial MMP-2: role in vascular smooth muscle cell migration. *Biochemical and Biophysical Research Communications*.

[30] Lévy M., Maurey C., Celermajer D. S. (2007). Impaired apoptosis of pulmonary endothelial cells is associated with intimal proliferation and irreversibility of pulmonary hypertension in congenital heart disease. *Journal of the American College of Cardiology*.

[31] Catalán Ú., Fernández-Castillejo S., Pons L. (2012). Alpha-tocopherol and BAY 11-7082 reduce vascular cell adhesion molecule in human aortic endothelial cells. *Journal of Vascular Research*.

[32] Eleftheriadis T., Antoniadi G., Liakopoulos V., Pissas G., Stefanidis I., Galaktidou G. (2012). Plasma angiogenin and vascular endothelial growth factor a among hemodialysis patients. *Iranian Journal of Kidney Diseases*.

[33] Cagnin S., Biscuola M., Patuzzo C. (2009). Reconstruction and functional analysis of altered molecular pathways in human atherosclerotic arteries. *BMC Genomics*.

[34] Chen S. C., Lin Y. L., Huang B., Wang D. L., Cheng J. J. (2011). Salvianolic acid B suppresses IFN-*γ*-induced JAK/STAT1 activation in endothelial cells. *Thrombosis Research*.

[35] Bredie S. J. H., Wollersheim H., Verheugt F. W. A., Thien T. (2003). Low-dose aspirin for primary prevention of cardiovascular disease. *Seminars in Vascular Medicine*.

[36] Wilson R., Gazzala J., House J. (2012). Aspirin in primary and secondary prevention in elderly adults revisited. *Southern Medical Journal*.

[37] Khan S. A., Ali A., Khan S. A. (2014). Unraveling the complex relationship triad between lipids, obesity, and inflammation. *Mediators of Inflammation*.

[38] Laakso M., Kuusisto J. (2014). Insulin resistance and hyperglycaemia in cardiovascular disease development. *Nature Reviews Endocrinology*.

[39] Hotamisligil G. S. (2010). Endoplasmic reticulum stress and the inflammatory basis of metabolic disease. *Cell*.

[40] Tjokroprawiro A. (2006). New approach in the treatment of T2DM and metabolic syndrome (focus on a novel insulin sensitizer). *Acta Medica Indonesiana*.

[41] Hur K. Y., Seo H. J., Kang E. S. (2008). Therapeutic effect of magnesium lithospermate B on neointimal formation after balloon-induced vascular injury. *European Journal of Pharmacology*.

[42] Wu X.-J., Wang Y.-P., Wang W., Sun W.-K., Xu Y.-M., Xuan L.-J. (2000). Free radical scavenging and inhibition of lipid peroxidation by magnesium lithospermate B. *Acta Pharmacologica Sinica*.

[43] Ba J., Peng H., Chen Y., Gao Y. (2014). Effects and mechanism analysis of vascular endothelial growth factor and salvianolic acid B on 125I-low density lipoprotein permeability of the rabbit aortary endothelial cells. *Cell Biochemistry and Biophysics*.

[44] Joe Y., Zheng M., Kim H. J. (2012). Salvianolic acid B exerts vasoprotective effects through the modulation of heme oxygenase-1 and arginase activities. *Journal of Pharmacology and Experimental Therapeutics*.

[45] Yang T.-L., Lin F.-Y., Chen Y.-H. (2011). Salvianolic acid B inhibits low-density lipoprotein oxidation and neointimal hyperplasia in endothelium-denuded hypercholesterolaemic rabbits. *Journal of the Science of Food and Agriculture*.

[46] Chen Y.-H., Du G.-H., Zhang J.-T. (2000). Salvianolic acid B protects brain against injuries caused by ischemia-reperfusion in rats. *Acta Pharmacologica Sinica*.

