# Supplementary material for: Significant Modules and Biological Processes between Active Components of Salvia miltiorrhiza Depside Salt and Aspirin
Source: Evid Based Complement Alternat Med. 2016 Mar 16;2016:3439521. doi: 10.1155/2016/3439521 (PMC4812280; doi:10.1155/2016/3439521)
Supplement: Supplementary file 1 — There are 5 tables in the Supplementary Material file. Table 1 contained the detailed genes related to active components of S. miltiorrhiza depside salt and aspirin. Table 2 and table 3 contained the modules of active components of S. miltiorrhiza depside salt and aspirin network, and ever modules' related genes. Table 4 and table 5 contained the detail of every biological function and KEGG pathway related to active components of S. miltiorrhiza and aspirin. [file 3439521.f1.pdf]

Table 1

Genes related to active compounds of salvianolate and aspirin, overlapping genes of them

**Genes related to active compounds of salvianolate**	JUN，NOS3，AKR1B1，NOS2，SOD1，PTGER3，PTGER2，MMP2，VCAM1，CASP3，MAPK8，IFNG，TNF，MMP1，EDN1，SIRT1，SHC1，SIK2，SERPINE1，MAPK1，TGFB1，ATF4，ATF6，APP，XDH，PTPN1，SPTLC2，PLOD1，SPTLC1，SLC16A1，ERVK-6，ALOX5，CCL11，DYT10，LCK，CXCL2，PTGS2，CCR3，FOS，IL1B，ITK，PLCG1，CREB1，IL8，BCL2，ZAP70，NFKB1，MAP2K1，IKBKB，RELA，GRB2，EEF1A1，IL2，FYN，MCL1
**Aspirin-related genes**	PTGER2，TBX21，PTGS1，CACNG6，RGS7BP，COL26A1，LTC4S，P2RY12，SELP，CPOX，PTGS2，COX5A，FPR2，RNASE3，GP1BA，P2RY1，UGT1A6，ALOX5，APOH，CYSLTR1，CANT1，PECAM1，PPBP，MS4A2，TBXA2R，F3，DUSP2，TFF2，TNXA，ITGB3，VASP，MT-CO1，ACE，ALOX12，ALOX15，CCL24，CYSLTR2，DPCR1，GP5，PF4，TFF3，F2，F2R，FGA，CYP2C9，GP6，ADH7，HNMT，PTGDR，SULT1A3，HLA-DPB1，ODC1，CEP68，F12，PTGDR2，SELE，CD40LG，KNG1，CRP，ALOX5AP，CD63，IGES，MYBPH，PTGIS，SAA4，BCHE，SERPINC1，ITGA2，ITGA2B，F2RL3，FCER1G，FCER1A，P2RX1，PAFAH1B2，PTGER1，TFF1，AHSP，ASIC3，CCL11，CES2，DEFA1，FCRL6，GGT2，GORASP1，GUSB，IL17D，LILRB4，OXA1L，PAFAH1B3，PEAR1，SOX7，THBD，TRIM26，UGT1A，VWF，WDR46，ZNRD1，C1S，HRH2，PTGER4，F10，G6PD，SCARB1，PLAT，SERPINE1，IL5，CTNNB1，IGHE，IL13，IL3，CASP1，TYMP，ADAM33，CEACAM7，CLDN2，CLDN3，CLDN5，CLDN7，CLEC4A，CSF1，JMJD6，KIFC1，LPA，NAB1，SERPINA7，SMAP2，ADRA2A，CCL26，CYLD，ENPP3，F5，OLR1，SERPINB5，ADRB2，AGTR1，BIRC5，HMOX1，ITGAM，NOS3，ADA，BID，PON1，ALOX15B，CAMP，CD151，CD58，CTSG，DPEP1，GSTP1，HSPA4，IL17RA，IL1RN，KIF3A，KLF6，LSP1，LTB4R，MLH1，MMRN1，MYLK3，OPRL1，PNOC，POSTN，PTGES2，REN，SGCB，SLC6A12，SNX3，TAPBP，TNFSF10，TNNI3，UGDH，VKORC1，ADORA1，ADORA3，ATP2A3，CCR3，CD59，CEBPB，CGB5，GAST，GRP，HADHA，HP，MCL1，NFKBIB，PPIG，SELL，VTN，NFKBIA，IL1B，IL4，NOS2，CCL2，ICAM1，IRS1，MMP2，PLA2G6，ABCA1，CD79A，CYP2C19，HMGCR，HNF4A，MPO，PLAU，PTGER3，RPS27A，TBXAS1，TIMP1，VCAM1，ANXA5，AP2B1，CCK，ELANE，FSIP1，HDAC3，IL2RB，ITGAL，PLAUR，PPARD，SERPINA1，ADAMTS13，ADORA2B，ADORA2A，AIFM1，ANXA4，CASP9，CCL22，CNTF，COG2，CYBB，DCBLD2，DDIT3，DIABLO，EDN1，ENPP2，F7，GFPT1，GTF2H4，HLA-DRB4，HPGD，IDO1，IL11，IL1R2，IL5RA，LAMP2，LOX，LTA4H，MPL，MTHFR，NLRP3，PCNA，PLEK，PNPLA8，PTGDS，PTGES，PTGIR，PTGFR，SAT1，SLC22A2，SLC26A5，SLPI，SSTR2，SULT1A1，TAP1，TAP2，TTF2，UGT2B7，JUN，MAPK14，TGFB1，TNF，CALCA，CCL5，CGA，CSF2，F13A1，F8，IL18，LDLR，MMP1，NGF，NPPB，POMC，TTR，ACP1，AKR1B1，ANPEP，BDKRB2，C6，CD9，CES1，CHKB，DDR1，FGB，GBA，GC，H2AFX，HLA-DPA1，HLA-DRB1，IL10，IL17A，LGALS3，NAT2，PAM，PDE4A，PLA2G7，STAT6，STK10，TGIF1，THPO，UBE3C，VIP，ADAMTSL1，APOB，BAX，DYT10，FAS，FN1，IGFBP3，IKBKB，KLK3，PLA2G2A，PPARA，VDR，IL6，APOE，CASP3，CDH1，IL1RAPL2，PPARG，A2M，ABCB11，ADAM17，CD40，CETP，CNR1，CP，CYP1A2，DTD1，FANCC，GGT1，HRH1，HSPA5，IFNG，IL15，IL8，IRS2，MAP4K4，NQO1，OBSCN，OXT，PEPD，PIK3C2A，PNPLA3，PPP2R4，PRKAA2，PTPN6，RPS6KA3，SLCO1B1，TNFRSF11B，TRAF6，ADIPOQ，APC，APOA1，ATM，CALM1，CAT，CFTR，CHUK，

	EPO，HGF，HLA-DQB1，IL2RA，KITLG，NOS1，TGFA，TLR4，BCL2，ACHE，ADCY10，AGT，ALB，ANGPT1，CAV1，CDK2，CDK4，CYP1A1，CYP3A4，FGF1，GFAP，HBB，HLA-C，HLA-DQA1，IFNA2，IL1A，IL1R1，JAK1，MAPK1，NTRK1，SLC2A1，TKT，TYR，XDH，CCND1，MAPK8，MMP9，NFKB1，ABCB1，EGF，ERBB2，ADCY1，AR，BCL2L1，BMP6，CD36，CSF3，CXCR4，DNAH8，HSPG2，ITGB1，JAK2，LIPC，LPL，PARP1，PIK3CA，PIK3CG，PLA2G4A，PTK2B，RELA，STAT1，TCF4，TP53，EGFR，VEGFA，BRCA1，BRAF，CDKN1A，HLA-B，RAF1，RPS6KA2，SOD1，SRC，STAT3，APP，KCNMA1，INS，IGF1，AKT1，CASP8，TNFRSF10B，OMA1，DIF，PLG，THBS1，LDLCQ3，LPO，FTL，FTH1，SLC22A7，CYP2B6，BDNF，SCARB2，HIF1A，TRH，EIF2AK3，AKR1C1，RECK，SLC22A6，CYP2C8，SP1，PRKAB1，CEBPG，RAC1，TNFRSF10A，MAPK3，HSF1，CASP6，GSK3B，BBC3，AKR1C2，AKR1C3，UGT1A8，UGT1A7，UGT1A10，PRKCD，PMAIP1，SLC22A11，ILK，CASP7，DCD，SLC22A9，TF，LTF，EPX，REL，MFI2，FPR1，GALC
**Overlapping genes**	JUN，NOS3，AKR1B1，NOS2，SOD1，PTGER3，PTGER2，MMP2，VCAM1，CASP3，MAPK8，IFNG，TNF，MMP1，EDN1，SERPINE1，MAPK1，TGFB1，APP，XDH，ALOX5，CCL11，DYT10，PTGS2，CCR3，IL1B，IL8，BCL2，NFKB1，IKBKB，RELA，MCL1

Two overlapping modules of active compounds of salvianolate's and aspirin's network

Modules	Genes	GOTERM_BP_ALL		KEGG_PATHWAY	
		Term	P-value	Term	P-value
$M_{(s3a10)}$	spn, cd97, cd37, icoslg, tnfsf9, emr2, cd86	GO:0002697~regulation of immune effector process	1.00E-02	Cell adhesion molecules (CAMs)	3.90E-03
		GO:0050776~regulation of immune response	2.30E-02	Intestinal immune network for IgA production	3.80E-02
		GO:0002684~positive regulation of immune system process	2.80E-02		
		GO:0002682~regulation of immune system process	3.90E-02		
		GO:0048583~regulation of response to stimulus	4.00E-02		
$M_{(s33a114)}$	fasn, dgat2, scd	GO:0008610~lipid biosynthetic process	3.20E-04	None	
		GO:0044255~cellular lipid metabolic process	9.10E-04		
		GO:0006629~lipid metabolic process	2.00E-03		
		GO:0006633~fatty acid biosynthetic process	1.20E-02		
		GO:0046394~carboxylic acid biosynthetic process	2.00E-02		
		GO:0016053~organic acid biosynthetic process	2.00E-02		
		GO:0044249~cellular biosynthetic process	2.20E-02		

		GO:0006631~fatty acid metabolic process	2.40E-02		
		GO:0009058~biosynthetic process	2.40E-02		
		GO:0032787~monocarboxylic acid metabolic process	3.40E-02		

Table 2

Modules of active compounds of salvianolate's network

Modules	Score	Nodes	Edges	Node IDs
1	15	15	105	il10, mmp9, mmp12, tnfa, mmp1, gc, adam33, timp2, mmp2, timp3, serpina1, serpina3, mmp3, il8, lta
2	9.455	45	208	nampt, il17ra, blk, ifna5, btk, ifna6, sirt1, blnk, ifna8, lat2, ifna14, irs2, mapk14, il2, dok1, jun, il13, spry1, ifng, spry2, stam2, zap70, shc1, kdr, frap1, vegfa, lat, bax, lcp2, sh2d2a, grb2, bcl2, akt1, pik3ca, prkaa1, ephb2, ros1, il17a, mmp7, lyn, syk, src, vav1, pln, mapk8
3	7	7	21	emr2, tnfsf9, cd86, icoslg, cd37, cd97, spn
4	6.444	19	58	stat1, smad2, f9, rela, ccl2, osm, myc, timp1, pold3, cdkn2a, igf1, igf1r, csf1, ccl5, tp53, ccl3, csf3, ccl11, sod1
5	6	6	15	slc16a1, slc16a3, slc16a7, slc16a8, cma1, slc16a4
6	5	5	10	eng, mcam, itgam, ptprc, cd34
7	5	5	10	actb, il1rn, il1r2, pgk1, il1b
8	4	4	6	cdc25c, ifi27, cdk2, cdkn1a
9	4	4	6	ptgfrn, esr2, gja1, ptger4
10	4	4	6	cd68, snai2, fap, actin
11	4	4	6	cxcr4, par1, itk, mirn200b
12	4	4	6	ikbkb, tcf4, lin28b, tcf7l2
13	3.911	46	88	zbtb16, clec10a, mcm8, zbtb32, cdk5, dynamin, crmp1, fyn, sp7, eif2ak3, ibsp, bcr, cd4, crtc2, crtc3, atf4, hdac4, hspa5, ccnh, ryr1, bcl2l11, atf6, apaf1, mlc1, cd2, ervk2, chek1, tnfsf13, f2rl1, egfr, bad, cdc7, cdk9, hspa4, hcc, hsf1, lck, uts2r, hsr, eef1a1, creb1, rgs2, pgr, cd8a, map2k1, runx2
14	3.714	8	13	tlr4, zc3h12a, tank, angpt1, il1r1, usp10, irak1, irf6
15	3.333	4	5	tyrp1, trpc6, itpr1, trpm4
16	3.333	4	5	spic, nfkb1, spi1, spib
17	3	3	3	setd2, srpk1, eif4e
18	3	3	3	eif4g2, vcp, 3.6.1.8
19	3	3	3	smad3, mid1, cemp1
20	3	3	3	muc1, muc3a, mmp13
21	3	3	3	cdc2, mapk3, mcl1
22	3	3	3	ppig, nqo1, cyp2b6
23	3	3	3	cxcl10, cx3cl1, cxcl11
24	3	3	3	arhgef2, icam1, nppa
25	3	3	3	camk1, hdac5, mef2c
26	3	3	3	crebbp, ep300, hdac6
27	3	3	3	plod1, plod2, mmrn1

28	3	3	3	visa, tmem173, trim69
29	3	3	3	lilrb4, slamf7, cr1
30	3	3	3	tnf, ikbkg, traf6
31	3	3	3	kat5, ptger3, camp
32	3	3	3	tcdd, pah, stat5a
33	3	3	3	fasn, scd, dgat2
34	3	3	3	cd22, cd79a, cd79b
35	3	3	3	rb1, s100a6, ccnd1
36	3	3	3	s100a8, s100a12, s100a9
37	3	3	3	cd28, ddx41, th1l
38	3	3	3	slc3a2, ppia, bsg

Table 3

Modules of aspirin's network

Modules	Score	Nodes	Edges	Node IDs
1	22.115	88	962	tgfbi, icam1, cd40lg, faslg, tnc, itga5, itgb2, col5a1, akt1, col11a1, fn1, adamts8, mmp8, ctnnd2, tp53, clec3b, cdkn1a, timp1, hmi, bbc3, birc3, birc8, bnip1, casp7, cox2, casp9, cd5, col4a3, znf443, utp11l, tp73, itga6, tnfrsf1b, tnfrsf25, tiaf1, stk17a, sst, spp1, cd44, nr3c1, tnf, il17a, cebpg, runx1, sparc, ctgf, ndufa5, ndufb10, ndufs1, sdha, uqcrh, adamts1, cox5a, cox5b, il22, atp5a1, ccl20, atp5b, rorc, atp5h, il26, dlst, il23r, idh2, ccr6, idh3a, ceacam1, ogdh, mcam, tnfsf10, aldoa, cd28, eno1, tnfsf13b, fbp1, ptpn13, gpi, map3k4, pgk1, ltb, taldo1, ctsh, atf5, arntl, cxcr6, furin, itgal, mmrn1
2	19	19	171	cbx5, jmjd2a, rcor1, ezh2, sin3a, hdac2, mbd1, jmjd3, ptger4, aof2, ptger2, dnmt3a, dnmt1, hdac1, hdac3, ep300, mecp2, dnmt3b, suv39h1
3	14.847	86	631	hspb1, pcsk1, csf1r, socs3, irf6, mmp9, vcam1, gstp1, flt3, lta, crp, lbp, il10, slc10a1, slco2b1, slc22a7, abcb11, abcc2, abcc3, bcr, abcc4, slco1b1, slco1b3, abcg2, slc22a1, cyp2b6, cyp2d6, uqcrfs1, cyp1a2, cyp2e1, cyp2c19, cyp2c9, fos, ptk2b, jak2, mmp13, tnfa, gc, adam33, timp2, timp3, serpina1, serpina3, mmp1, crlf2, mmp3, ntrk3, ccl7, tyk2, il7r, cyp19a1, sh2b3, mmp2, abl2, cyp1b1, pdgfrb, epor, elf4, il4, nat2, jun, gstm1, gstm3, gsto1, pon1, pon2, avp, ccl2, cxcl10, mmp12, il6, cyp1a1, abl1, tslp, tnfaip3, nfkbia, egr1, enpp2, hk2, mmp10, gadd45a, wisp1, cebpb, selplg, bcl2a1, vegfa
4	9.586	112	532	krt7, apoa4, krt8, apob, krt19, apoc3, ttr, ervk2, msmb, apoh, cdkn2a, ptgs2, mlh1, apba1, apom, apba2, dapk1, rassf1, brca1, src, cftr, gata1, cux1, il17ra, bcl2l1, krt16, mki67, cd1b, apoe, cd1c, ccl22, ccl26, nos2, plaur, ifng, pou5f1, nanog, abcb6, actin, tnfrsf10b, ddit3, etv4, mapk14, nfe2l1, mapk8, tbx6, tnfsf13, tbx5, myf5, hla-e, ephb2, nkx2-5, tnnt2, kdr, tgfb, plau, pax7, ncam1, xiap, ptk2, cd40, pik3ca, f2r, bax, ccl18, a2m, egfr, mark2, cyp24a1, cd14, slc1a1, pou2f1, slc22a3, tap2, thbd, plat, procr, smad3, gpx1, gpx4, gata3, sepw1, sepn1, hla-b, ccl17, b2m, psmb9, psmb8, tap1, ppa1, ulbp2, ulbp3, diablo, col9a1, tapbp, cd34, th1l, nfat5, f2, cldn7, atp8a2, tymp, stat1, mmp7, hspa5, itgb3, klf4, csf3,

				csf3r, eef1b3, apoa1, apoa2
5	9	9	36	vhl, iqsec1, foxp1, lrrn1, gnai2, fgd5, aldh1l1, bcl6, ctdspl
6	8	8	28	slc39a8, c3ar1, fcgr3b, rad21, retn, lrrc25, slc3a2, sco1
7	7.92	26	99	slc35a2, nqo1, ugt1a1, sod2, nfe2l2, selp, gabpa, ros1, hmox1, scarb1, s1pr2, s1pr3, pah, il1b, hsd11b1, ugt1a7, abca1, s1pr1, mbtps1, ugt1a10, ugt1a3, ugt1a6, ugt1a9, ugt2b7, ugt2b15, ugt1a4
8	7	7	21	flt1, phactr1, psrc1, cbs, lpl, cnnm2, cdkn2b
9	7	7	21	cgref1, pa2g4, ppp2r1b, basp1, plec1, itgb4, lama5
10	7	7	21	emr2, tnfsf9, icoslg, cd37, cd97, spn, cd86
11	6.625	33	106	c6orf136, ccl25, xcl2, ccl19, il2, foxm1, ccl21, csf1, il8ra, igf1, ifna5, ifna6, ifna8, il8, ifna14, ptgfr, fstl1, ifi16, nampt, sell, sele, igf1r, ccl5, spcs1, flna, chpf, mgat1, ndufa10, pafah1b3, ifna1, ccl11, glt8d1, ccl24
12	6	6	15	plekhb2, fcgbp, ubd, dbndd2, syt1, znf560
13	5.467	16	41	tnni1, myh2, myl1, myh3, idh1, myh14, lcp1, mybph, mpeg1, mpo, itga9, pdlim5, limk1, rock1, 3.6.4.1, nkx2-1
14	5.333	10	24	cish, ace2, nfkbil1, ggt1, agt, agtr2, mas1, sry, sox3, aga
15	5.25	25	63	gfap, cox8a, fgl2, f2rl1, f2rl2, f2rl3, snx2, snx4, snx6, snx8, anxa7, adrb2, tjp1, snx9, snx21, cdc42, rac1, snx15, rhoa, snx5, snx3, mtg1, chn2, arhgap1, tbk1
16	5	5	10	clu, apol1, c9orf3, apod, apof
17	5	11	25	lif, pias3, il6st, soat1, epo, egr2, il11, thpo, il17d, kitlg, cxcl9
18	5	5	10	tac1, grb7, map3k15, klf7, nkx2-2
19	5	5	10	tlr2, hpgd, s100a8, s100a1, cbr1
20	5	5	10	ttf2, fbxo11, ppp1r10, wdr82, tox3
21	5	5	10	cfh, rara, cd55, il33, efemp1
22	5	5	10	pak2, dlg1, hes1, eif4g2, mfi2
23	5	5	10	krt5, ets2, ebp, krt14, ets1
24	5	5	10	ctcf, pax3, tbx1, myh7b, obscn
25	5	5	10	masp1, tfpi, c1r, c1s, masp2
26	4.667	10	21	setd2, srf, sp1, klf6, apcs, ebf1, mrxs5, sp3, gata4, ugdh
27	4.667	79	182	flt4, parp1, orc1l, mcm7, cdc6, orc2l, mcm3, il1a, dtd1, oit3, fgfr2, e2f5, sox9, gsk3b, bub1, ctnnb1, ccnb2, nf-kappab, f9, cldn3, mybl2, pparg, cdc20, bcl2, hspg2, aph1b, map3k7, psen1, psenen, ppig, cdh2, ncstn, snai2, mapk3, psen2, zeb1, myc, gli2, p2ry1, heca, p2ry12, lrp1, gp6, bcl2l2, par1, par4, smad2, ahr, nod2, tnfsf15, mapk9, tnfrsf4, mcl1, stat6, ptpn11, msr1, ppp2r4, map2k4, srpk1, eif4e, kras, igfals, mrc1, msh2, zhx2, pms2, 2.7.7.48, rela, cdc2, frap1, decr1, casr, itk, fyn, rage, atm, wdtc1, cd36, vegfc
28	4.333	7	13	cd79b, nol3, igkv2-23, pnoc, hars, rln2, s100a4
29	4.16	26	52	muc3a, muc5ac, il13, atf3, adrbk1, inppl1, dio2, grb2, tbxa2r, fcrl6, osm, lair1, inpp5d, akt3, jak1, ptpn6, jag1, lilrb3, sirpa, cd200r1, mafa, cd72, nos3, stat3, nfkbil2, tgfb3
30	4	4	6	aoc3, lipe, sod3, aqp7
31	4	4	6	ccnb1, c9orf66, pmp22, opcml
32	4	4	6	galr1, slc5a8, agtr1, zmynd10
33	4	4	6	zfp36, khsrp, pabpc1, elavl1
34	4	4	6	cdk5, stxbp3, fcn2, cdk5r2
35	4	4	6	h2afy, ctsa, il5ra, rb1

36	4	4	6	foxp3, lrba, ikzf2, il2ra
37	4	4	6	ndufa9, ndufs4, ndufv2, adcy10
38	4	4	6	ing3, ckap2, ptk6, rps6ka1
39	4	4	6	padi2, padi3, padi4, aifm1
40	4	4	6	mipep, yme1l1, oxa1l, mrs2
41	4	4	6	wdr46, ddx21, nop2, ebna1bp2
42	4	4	6	pdgfra, lrp8, igfbp5, ddit4
43	4	4	6	chtf18, nhp2l1, app, znhit3
44	4	4	6	wdr5, mll3, utx, piwil1
45	4	4	6	foxg1, grik3, hand2, prkcb
46	4	4	6	elovl2, fhl2, klf14, glra1
47	3.875	17	31	cxcr4, sema3f, ascl2, mirn200b, apobec3c, skp2, apobec3d, col4a2, cd4, ctr9, spry1, agrp, trim26, mirn29a, trim32, mirn21, reck
48	3.778	10	17	vdr, cyp11a1, cyp17a1, trak1, nrip1, gclc, hsd17b6, gpc3, dusp10, runx2
49	3.692	14	24	ntrk1, ntsr1, snrpg, itgav, sqstm1, lamb1, tnfsf11, itgb1, slurp1, ngfr, ngf, ern1, calm1, nfatc2
50	3.6	6	9	traf6, angpt1, irak1, tank, usp10, zc3h12a
51	3.375	17	27	cdk2, lims1, ipp, rsu1, srebf2, hmgcr, ifi27, alox5, cdkn1b, cdc25c, bak1, pmaip1, fas, cox1, alox15b, alox12, ldlr
52	3.375	17	27	mapk1, ripk3, aaas, bcatenin, hnf1a, dym, bcl9l, wwox, csnk1a1, dvl2, nedd9, bcar1, myo18b, hoxc6, kif3a, prkab1, agfg1
53	3.375	17	27	hrh1, hsp90aa1, pkc, appl1, raf1, iqgap1, rab5a, ilk, tgfa, kcnj10, map2k1, erbb2, fgf2, egf, neurog2, ascl1, dag1
54	3.333	4	5	pdf, aqp4, cd46, cd59
55	3.333	4	5	adora2a, adora2b, nt5e, igkv2d-29
56	3.333	4	5	stk24, slk, stk10, plk1
57	3.333	7	10	en1, cdh13, anapc11, ube2c, apc2, cdh1, hrh2
58	3.143	15	22	cd244, cysltr1, cysltr2, nfkb1, slc45a2, alox5ap, anxa1, ubxn1, ugt1a@, cyp3a4, uts2r, slco1a2, slc22a6, slc22a8, slc22a2
59	3.111	10	14	braf, irs2, hras, tbc1d4, itih5, cx3cl1, ret, mtch2, fto, irs1
60	3	3	3	cyp3a, cyp2a6, cyp2c8
61	3	3	3	ccr10, cx3cr1, cd69
62	3	3	3	mc2r, mc1r, mc5r
63	3	3	3	fgf1, fgfr1, mmp26
64	3	3	3	trpc6, sphk1, smpd2
65	3	3	3	afg3l1, oma1, opa1
66	3	3	3	serpinf2, s100a6, cxadr
67	3	3	3	epha3, pafah1b2, kiaa0101
68	3	3	3	fkbp5, klk3, tmprss2
69	3	3	3	foxp2, kcnq1, hdac4
70	3	3	3	tg, picalm, smap2
71	3	3	3	chek1, cdc7, cdk9
72	3	3	3	lig3, mirn22, mirn150
73	3	3	3	hla-a29.1, anpep, hdc
74	3	3	3	il1rn, il1r2, actb
75	3	3	3	gja1, ptgfrn, esr2
76	3	3	3	pecam1, cd300a, preb

77	3	3	3	xrcc1, hgf, dusp2
78	3	3	3	sim2, per1, arnt
79	3	3	3	dnajc3, dnajb6, hsp90b2p
80	3	3	3	ptx3, rhob, rhoc
81	3	7	9	tlr4, sarm1, foxo1, abhd5, nucb2, nlrp3, myd88
82	3	3	3	pi3, tys, gpsn2
83	3	3	3	klk4, sds, klk2
84	3	3	3	mapkapk2, fanca, fancc
85	3	3	3	baz1b, dgcr14, rps6ka3
86	3	3	3	mirn130a, acvr1, bmp6
87	3	3	3	traf3, smpd1, pde4a
88	3	3	3	ikzf3, zpbp2, tmem39a
89	3	3	3	gorasp1, gorasp2, grasp
90	3	3	3	jmjd5, hif1an, jmjd6
91	3	3	3	afm, atrn, fbn1
92	3	3	3	gas5, snord15a, mll
93	3	3	3	adar, adarb1, adarb2
94	3	3	3	ldhb, fat, cdkn2d
95	3	3	3	cep68, triobp, trio
96	3	3	3	ctnnd1, pak6, anxa5
97	3	3	3	ndufc2, thada, mef2a
98	3	3	3	tcf7l2, ikbkb, lin28b
99	3	3	3	neurog1, aldh7a1, gria1
100	3	3	3	dgat2, fasn, scd
101	3	3	3	oxtr, rpe, oxt
102	3	3	3	rheb, tsc2, mcrs1
103	3	3	3	ptpn22, acp1, zap70
104	3	3	3	myt1l, zranb1, nploc4
105	3	3	3	spon1, pofut2, cfp
106	3	3	3	ranbp1, gsta1, gsta2
107	3	3	3	ndel1, pafah1b1, adra1d
108	3	3	3	defa1, defa3, defb1
109	3	3	3	aldh1a1, rgs19, oprl1
110	3	3	3	fbxo25, fh, hax1
111	3	3	3	fgg, fga, fgb
112	3	3	3	3.4.13.3, nt5c1a, cndp2
113	3	3	3	dmrt1, slc39a14, c4bpa
114	3	3	3	mt1l, mbp, lamc2
115	3	3	3	cntf, ctnnbl1, rnase3
116	3	3	3	bp8, adh7, rdh10
117	3	3	3	cfb, plp2, saa4
118	3	3	3	rps6kb1, prkaa2, arg2
119	3	3	3	pvr, kng1, igfbp6
120	3	3	3	1.2.2.2, ppm2c, pdk4
121	3	3	3	casp6, nlrp1, casp1
122	2.667	7	8	serpina7, aavs1, ass1, apc, cd1a, cd207, lcs1

Table 4

762 GO biological functions and 63 KEGG pathways in top 10 non-overlapping modules of active compounds of salvianolate's network

Modules	Genes	GOTERM_BP_ALL		KEGG_PATHWAY	
		Term	P-value	Term	P-value
$M_{(s1)}$	il10, mmp9, mmp12, tnfa, mmp1, gc, adam33, timp2, mmp2, timp3, serpina1, serpina3, mmp3, il8, lta	GO:0030574~collagen catabolic process	1.50E-05	Bladder cancer	7.60E-06
		GO:0044243~multicellular organismal catabolic process	3.60E-05	Pathways in cancer	4.00E-03
		GO:0032963~collagen metabolic process	5.50E-05	Cytokine-cytokine receptor interaction	1.90E-02
		GO:0044259~multicellular organismal macromolecule metabolic process	6.60E-05		
		GO:0044236~multicellular organismal metabolic process	1.20E-04		
		GO:0051045~negative regulation of membrane protein ectodomain proteolysis	2.10E-03		
		GO:0006508~proteolysis	2.70E-03		
		GO:0042177~negative regulation of protein catabolic process	5.30E-03		
		GO:0045861~negative regulation of proteolysis	6.30E-03		
		GO:0051043~regulation of membrane protein ectodomain proteolysis	7.40E-03		
		GO:0031330~negative regulation of cellular catabolic process	8.50E-03		
		GO:0032501~multicellular organismal process	9.20E-03		
		GO:0009895~negative regulation of catabolic process	1.40E-02		
		GO:0030162~regulation of proteolysis	1.90E-02		
		GO:0042176~regulation of protein catabolic process	2.20E-02		
		GO:0031329~regulation of cellular catabolic process	2.40E-02		
		GO:0009894~regulation of catabolic process	3.70E-02		
		GO:0006955~immune response	3.70E-02		
		GO:0040017~positive regulation of locomotion	3.80E-02		
		GO:0048731~system development	4.70E-02		
$M_{(s2)}$	nampt, il17ra, blk, ifna5, btk,	GO:0007243~protein kinase cascade	4.00E-11	Fc epsilon RI signaling pathway	4.60E-12

	ifna6, sirt1, blnk, ifna8, lat2, ifna14, irs2, mapk14, il2, dok1, jun, il13, spry1, ifng, spry2, stam2, zap70, shc1, kdr, frap1, vegfa, lat, bax, lcp2, sh2d2a, grb2, bcl2, akt1, pik3ca, prkaa1, ephb2, ros1, il17a, mmp7, lyn, syk, src, vav1, pln, mapk8	GO:0018193~peptidyl-amino acid modification	4.10E-11	Natural killer cell mediated cytotoxicity	9.40E-11
		GO:0006468~protein amino acid phosphorylation	4.20E-11	Jak-STAT signaling pathway	5.70E-10
		GO:0007167~enzyme linked receptor protein signaling pathway	2.60E-10	T cell receptor signaling pathway	3.50E-09
		GO:0048518~positive regulation of biological process	2.80E-10	B cell receptor signaling pathway	5.30E-08
		GO:0016310~phosphorylation	6.10E-10	Neurotrophin signaling pathway	2.00E-07
		GO:0007169~transmembrane receptor protein tyrosine kinase signaling pathway	1.00E-09	Toll-like receptor signaling pathway	5.50E-07
		GO:0045321~leukocyte activation	2.10E-09	Focal adhesion	1.30E-06
		GO:0048522~positive regulation of cellular process	2.20E-09	Regulation of autophagy	4.90E-06
		GO:0008284~positive regulation of cell proliferation	2.30E-09	Cytokine-cytokine receptor interaction	1.50E-05
		GO:0050896~response to stimulus	4.50E-09	VEGF signaling pathway	1.60E-05
		GO:0018108~peptidyl-tyrosine phosphorylation	4.80E-09	Colorectal cancer	3.10E-05
		GO:0042127~regulation of cell proliferation	4.80E-09	ErbB signaling pathway	3.80E-05
		GO:0018212~peptidyl-tyrosine modification	6.20E-09	RIG-I-like receptor signaling pathway	1.60E-04
		GO:0007242~intracellular signaling cascade	7.60E-09	Insulin signaling pathway	4.30E-04
		GO:0006793~phosphorus metabolic process	1.00E-08	Autoimmune thyroid disease	5.20E-04
		GO:0006796~phosphate metabolic process	1.00E-08	Fc gamma R-mediated phagocytosis	6.30E-04
		GO:0001775~cell activation	1.10E-08	Epithelial cell signaling in Helicobacter pylori infection	1.50E-03
		GO:0002376~immune system process	1.50E-08	Renal cell carcinoma	1.70E-03
		GO:0007166~cell surface receptor linked signal transduction	1.60E-08	GnRH signaling pathway	5.80E-03
		GO:0043687~post-translational protein modification	2.30E-08	mTOR signaling pathway	6.60E-03
		GO:0048513~organ development	3.30E-08	Cytosolic DNA-sensing pathway	7.70E-03
		GO:0007165~signal transduction	4.70E-08	Pathways in cancer	9.70E-03
		GO:0050793~regulation of developmental process	5.80E-08	Glioma	1.10E-02
		GO:0051704~multi-organism process	6.50E-08	Chemokine signaling pathway	1.20E-02
		GO:0006464~protein modification process	7.40E-08	Adipocytokine signaling pathway	1.30E-02

		GO:0048583~regulation of response to stimulus	9.80E-08	Progesterone-mediated oocyte maturation	1.60E-02
		GO:0042325~regulation of phosphorylation	1.00E-07	Apoptosis	1.80E-02
		GO:0006950~response to stress	1.20E-07	Primary immunodeficiency	2.30E-02
		GO:0051094~positive regulation of developmental process	1.20E-07	Prostate cancer	2.60E-02
		GO:0019220~regulation of phosphate metabolic process	1.50E-07	Type II diabetes mellitus	2.70E-02
		GO:0051174~regulation of phosphorus metabolic process	1.50E-07		
		GO:0043412~biopolymer modification	1.60E-07		
		GO:0032879~regulation of localization	1.70E-07		
		GO:0002682~regulation of immune system process	1.70E-07		
		GO:0045597~positive regulation of cell differentiation	3.80E-07		
		GO:0030154~cell differentiation	4.60E-07		
		GO:0030097~hemopoiesis	4.70E-07		
		GO:0051049~regulation of transport	5.40E-07		
		GO:0006955~immune response	6.40E-07		
		GO:0048869~cellular developmental process	8.50E-07		
		GO:0048731~system development	8.70E-07		
		GO:0048534~hemopoietic or lymphoid organ development	9.90E-07		
		GO:0032844~regulation of homeostatic process	1.20E-06		
		GO:0009966~regulation of signal transduction	1.20E-06		
		GO:0010646~regulation of cell communication	1.30E-06		
		GO:0045576~mast cell activation	1.50E-06		
		GO:0002520~immune system development	1.50E-06		
		GO:0045595~regulation of cell differentiation	1.60E-06		
		GO:0046649~lymphocyte activation	2.10E-06		
		GO:0051239~regulation of multicellular organismal process	2.50E-06		
		GO:0042981~regulation of apoptosis	3.20E-06		
		GO:0048856~anatomical structure development	3.40E-06		

		GO:0043067~regulation of programmed cell death	3.50E-06		
		GO:0010941~regulation of cell death	3.70E-06		
		GO:0051716~cellular response to stimulus	3.90E-06		
		GO:0050789~regulation of biological process	5.30E-06		
		GO:0002684~positive regulation of immune system process	6.80E-06		
		GO:0050794~regulation of cellular process	7.80E-06		
		GO:0006952~defense response	1.20E-05		
		GO:0043269~regulation of ion transport	1.20E-05		
		GO:0048872~homeostasis of number of cells	1.30E-05		
		GO:0009893~positive regulation of metabolic process	1.30E-05		
		GO:0001932~regulation of protein amino acid phosphorylation	1.30E-05		
		GO:0030098~lymphocyte differentiation	1.40E-05		
		GO:0042221~response to chemical stimulus	1.50E-05		
		GO:0007265~Ras protein signal transduction	1.60E-05		
		GO:0009615~response to virus	1.90E-05		
		GO:0050671~positive regulation of lymphocyte proliferation	2.30E-05		
		GO:0065007~biological regulation	2.40E-05		
		GO:0070665~positive regulation of leukocyte proliferation	2.40E-05		
		GO:0032946~positive regulation of mononuclear cell proliferation	2.40E-05		
		GO:0051707~response to other organism	2.50E-05		
		GO:0007275~multicellular organismal development	2.60E-05		
		GO:0032502~developmental process	3.20E-05		
		GO:0050790~regulation of catalytic activity	3.30E-05		
		GO:0009891~positive regulation of biosynthetic process	3.40E-05		
		GO:0022602~ovulation cycle process	3.70E-05		
		GO:0010604~positive regulation	3.70E-05		

		of macromolecule metabolic process			
		GO:0042110~T cell activation	3.80E-05		
		GO:0043065~positive regulation of apoptosis	3.90E-05		
		GO:0048511~rhythmic process	4.10E-05		
		GO:0043068~positive regulation of programmed cell death	4.10E-05		
		GO:0008585~femaleGOnad development	4.20E-05		
		GO:0010942~positive regulation of cell death	4.30E-05		
		GO:0002521~leukocyte differentiation	4.60E-05		
		GO:0031325~positive regulation of cellular metabolic process	4.70E-05		
		GO:0042698~ovulation cycle	5.00E-05		
		GO:0001101~response to acid	5.10E-05		
		GO:0051050~positive regulation of transport	5.40E-05		
		GO:0046660~female sex differentiation	5.60E-05		
		GO:0046545~development of primary female sexual characteristics	5.60E-05		
		GO:0050776~regulation of immune response	5.90E-05		
		GO:0032880~regulation of protein localization	5.90E-05		
		GO:0042592~homeostatic process	6.60E-05		
		GO:0009605~response to external stimulus	6.70E-05		
		GO:0048584~positive regulation of response to stimulus	7.40E-05		
		GO:0006928~cell motion	8.00E-05		
		GO:0051249~regulation of lymphocyte activation	8.30E-05		
		GO:0044267~cellular protein metabolic process	8.30E-05		
		GO:0002279~mast cell activation during immune response	9.00E-05		
		GO:0002448~mast cell mediated immunity	9.00E-05		
		GO:0043303~mast cell degranulation	9.00E-05		
		GO:0001568~blood vessel development	9.20E-05		
		GO:0043434~response to peptide	1.00E-04		

		hormone stimulus			
		GO:0001944~vasculature development	1.00E-04		
		GO:0010959~regulation of metal ion transport	1.00E-04		
		GO:0007154~cell communication	1.10E-04		
		GO:0008637~apoptotic mitochondrial changes	1.10E-04		
		GO:0065009~regulation of molecular function	1.10E-04		
		GO:0050670~regulation of lymphocyte proliferation	1.20E-04		
		GO:0070663~regulation of leukocyte proliferation	1.20E-04		
		GO:0032944~regulation of mononuclear cell proliferation	1.20E-04		
		GO:0010557~positive regulation of macromolecule biosynthetic process	1.30E-04		
		GO:0050851~antigen receptor-mediated signaling pathway	1.30E-04		
		GO:0050871~positive regulation of B cell activation	1.30E-04		
		GO:0002694~regulation of leukocyte activation	1.40E-04		
		GO:0009607~response to biotic stimulus	1.40E-04		
		GO:0001934~positive regulation of protein amino acid phosphorylation	1.50E-04		
		GO:0001776~leukocyte homeostasis	1.80E-04		
		GO:0031328~positive regulation of cellular biosynthetic process	1.80E-04		
		GO:0050865~regulation of cell activation	1.80E-04		
		GO:0051649~establishment of localization in cell	1.90E-04		
		GO:0008286~insulin receptor signaling pathway	1.90E-04		
		GO:0051251~positive regulation of lymphocyte activation	2.10E-04		
		GO:0042327~positive regulation of phosphorylation	2.10E-04		
		GO:0051272~positive regulation of cell motion	2.20E-04		
		GO:0002429~immune	2.20E-04		

		response-activating cell surface receptor signaling pathway			
		GO:0042102~positive regulation of T cell proliferation	2.20E-04		
		GO:0010562~positive regulation of phosphorus metabolic process	2.40E-04		
		GO:0045937~positive regulation of phosphate metabolic process	2.40E-04		
		GO:0032868~response to insulin stimulus	2.40E-04		
		GO:0009967~positive regulation of signal transduction	2.50E-04		
		GO:0031399~regulation of protein modification process	2.50E-04		
		GO:0019538~protein metabolic process	2.60E-04		
		GO:0001541~ovarian follicle development	2.60E-04		
		GO:0010033~response to organic substance	2.70E-04		
		GO:0033554~cellular response to stress	2.70E-04		
		GO:0002768~immune response-regulating cell surface receptor signaling pathway	2.80E-04		
		GO:0051270~regulation of cell motion	2.90E-04		
		GO:0002696~positive regulation of leukocyte activation	3.00E-04		
		GO:0050853~B cell receptor signaling pathway	3.20E-04		
		GO:0050867~positive regulation of cell activation	3.50E-04		
		GO:0045834~positive regulation of lipid metabolic process	3.60E-04		
		GO:0002274~myeloid leukocyte activation	3.60E-04		
		GO:0008406~gonad development	3.70E-04		
		GO:0051641~cellular localization	3.80E-04		
		GO:0006916~anti-apoptosis	3.90E-04		
		GO:0050731~positive regulation of peptidyl-tyrosine phosphorylation	3.90E-04		
		GO:0007267~cell-cell signaling	3.90E-04		
		GO:0006915~apoptosis	4.00E-04		
		GO:0050863~regulation of T cell activation	4.30E-04		
		GO:0048514~blood vessel	4.30E-04		

		morphogenesis			
		GO:0012501~programmed cell death	4.50E-04		
		GO:0010647~positive regulation of cell communication	4.50E-04		
		GO:0070201~regulation of establishment of protein localization	4.90E-04		
		GO:0050864~regulation of B cell activation	5.00E-04		
		GO:0032268~regulation of cellular protein metabolic process	5.20E-04		
		GO:0002757~immune response-activating signal transduction	5.20E-04		
		GO:0048608~reproductive structure development	5.70E-04		
		GO:0045859~regulation of protein kinase activity	5.80E-04		
		GO:0043299~leukocyte degranulation	5.90E-04		
		GO:0045137~development of primary sexual characteristics	5.90E-04		
		GO:0002764~immune response-regulating signal transduction	6.50E-04		
		GO:0051054~positive regulation of DNA metabolic process	6.50E-04		
		GO:0043066~negative regulation of apoptosis	6.60E-04		
		GO:0032270~positive regulation of cellular protein metabolic process	6.80E-04		
		GO:0043549~regulation of kinase activity	6.90E-04		
		GO:0043069~negative regulation of programmed cell death	7.10E-04		
		GO:0060548~negative regulation of cell death	7.20E-04		
		GO:0009411~response to UV	7.60E-04		
		GO:0009725~response to hormone stimulus	8.00E-04		
		GO:0033135~regulation of peptidyl-serine phosphorylation	8.10E-04		
		GO:0046632~alpha-beta T cell differentiation	8.10E-04		
		GO:0051247~positive regulation of protein metabolic process	8.20E-04		

		GO:0048878~chemical homeostasis 8	8.20E-04		
		GO:0051338~regulation of transferase activity	8.60E-04		
		GO:0043405~regulation of MAP kinase activity	8.70E-04		
		GO:0042129~regulation of T cell proliferation	8.80E-04		
		GO:0043085~positive regulation of catalytic activity	9.00E-04		
		GO:0001894~tissue homeostasis	9.20E-04		
		GO:0002444~myeloid leukocyte mediated immunity	9.30E-04		
		GO:0030217~T cell differentiation	1.00E-03		
		GO:0048754~branching morphogenesis of a tube	1.00E-03		
		GO:0001525~angiogenesis	1.00E-03		
		GO:0007548~sex differentiation	1.10E-03		
		GO:0003006~reproductive developmental process	1.10E-03		
		GO:0050730~regulation of peptidyl-tyrosine phosphorylation	1.10E-03		
		GO:0032869~cellular response to insulin stimulus	1.10E-03		
		GO:0006006~glucose metabolic process	1.20E-03		
		GO:0051246~regulation of protein metabolic process	1.20E-03		
		GO:0002053~positive regulation of mesenchymal cell proliferation	1.20E-03		
		GO:0051924~regulation of calcium ion transport	1.20E-03		
		GO:0032501~multicellular organismal process	1.30E-03		
		GO:0008219~cell death	1.30E-03		
		GO:0009719~response to endogenous stimulus	1.30E-03		
		GO:0046631~alpha-beta T cell activation	1.30E-03		
		GO:0045055~regulated secretory pathway	1.30E-03		
		GO:0010464~regulation of mesenchymal cell proliferation	1.30E-03		
		GO:0002275~myeloid cell activation during immune response	1.30E-03		
		GO:0016265~death	1.40E-03		
		GO:0001763~morphogenesis of a branching structure	1.50E-03		

		GO:0045058~T cell selection	1.50E-03		
		GO:0042113~B cell activation	1.60E-03		
		GO:0050870~positive regulation of T cell activation	1.60E-03		
		GO:0044419~interspecies interaction between organisms	1.60E-03		
		GO:0010676~positive regulation of cellular carbohydrate metabolic process	1.70E-03		
		GO:0045913~positive regulation of carbohydrate metabolic process	1.70E-03		
		GO:0048678~response to axon injury	1.70E-03		
		GO:0030334~regulation of cell migration	1.70E-03		
		GO:0044093~positive regulation of molecular function	1.80E-03		
		GO:0043029~T cell homeostasis	1.80E-03		
		GO:0046889~positive regulation of lipid biosynthetic process	1.80E-03		
		GO:0001836~release of cytochrome c from mitochondria	1.80E-03		
		GO:0008283~cell proliferation	1.90E-03		
		GO:0008633~activation of pro-apoptotic gene products	2.00E-03		
		GO:0048519~negative regulation of biological process	2.20E-03		
		GO:0048871~multicellular organismal homeostasis	2.20E-03		
		GO:0042531~positive regulation of tyrosine phosphorylation of STAT protein	2.20E-03		
		GO:0007264~small GTPase mediated signal transduction	2.20E-03		
		GO:0000165~MAPKKK cascade	2.30E-03		
		GO:0031401~positive regulation of protein modification process	2.50E-03		
		GO:0030335~positive regulation of cell migration	2.50E-03		
		GO:0055074~calcium ion homeostasis	2.50E-03		
		GO:0019318~hexose metabolic process	2.70E-03		
		GO:0040012~regulation of locomotion	2.70E-03		
		GO:0002637~regulation of immunoglobulin production	2.80E-03		
		GO:0046427~positive regulation	2.80E-03		

		of JAK-STAT cascade			
		GO:0002253~activation of immune response	2.90E-03		
		GO:0048468~cell development	3.10E-03		
		GO:0051173~positive regulation of nitrogen compound metabolic process	3.10E-03		
		GO:0007173~epidermal growth factor receptor signaling pathway	3.30E-03		
		GO:0046320~regulation of fatty acid oxidation	3.30E-03		
		GO:0002260~lymphocyte homeostasis	3.30E-03		
		GO:0040017~positive regulation of locomotion	3.30E-03		
		GO:0055065~metal ion homeostasis	3.40E-03		
		GO:0018105~peptidyl-serine phosphorylation	3.50E-03		
		GO:0010522~regulation of calcium ion transport into cytosol	3.50E-03		
		GO:0032940~secretion by cell	3.50E-03		
		GO:0002697~regulation of immune effector process	3.60E-03		
		GO:0021700~developmental maturation	3.60E-03		
		GO:0040008~regulation of growth	3.60E-03		
		GO:0009888~tissue development	3.70E-03		
		GO:0065008~regulation of biological quality	3.70E-03		
		GO:0080135~regulation of cellular response to stress	3.80E-03		
		GO:0042509~regulation of tyrosine phosphorylation of STAT protein	4.00E-03		
		GO:0060249~anatomical structure homeostasis	4.10E-03		
		GO:0001933~negative regulation of protein amino acid phosphorylation	4.20E-03		
		GO:0045582~positive regulation of T cell differentiation	4.20E-03		
		GO:0022603~regulation of anatomical structure morphogenesis	4.30E-03		
		GO:0009991~response to extracellular stimulus	4.40E-03		
		GO:0035295~tube development	4.40E-03		

		GO:0051047~positive regulation of secretion	4.40E-03		
		GO:0007568~aging	4.50E-03		
		GO:0005996~monosaccharide metabolic process	4.50E-03		
		GO:0045860~positive regulation of protein kinase activity	4.60E-03		
		GO:0008624~induction of apoptosis by extracellular signals	4.80E-03		
		GO:0019216~regulation of lipid metabolic process	4.80E-03		
		GO:0000060~protein import into nucleus, translocation	4.80E-03		
		GO:0009628~response to abiotic stimulus	5.00E-03		
		GO:0051052~regulation of DNA metabolic process	5.00E-03		
		GO:0051223~regulation of protein transport	5.00E-03		
		GO:0045621~positive regulation of lymphocyte differentiation	5.10E-03		
		GO:0007423~sensory organ development	5.10E-03		
		GO:0009611~response to wounding	5.10E-03		
		GO:0033674~positive regulation of kinase activity	5.20E-03		
		GO:0046425~regulation of JAK-STAT cascade	5.30E-03		
		GO:0002263~cell activation during immune response	5.30E-03		
		GO:0002366~leukocyte activation during immune response	5.30E-03		
		GO:0031331~positive regulation of cellular catabolic process	5.30E-03		
		GO:0019722~calcium-mediated signaling	5.60E-03		
		GO:0055066~di-, tri-valent inorganic cation homeostasis	5.90E-03		
		GO:0045767~regulation of anti-apoptosis	5.90E-03		
		GO:0010675~regulation of cellular carbohydrate metabolic process	5.90E-03		
		GO:0051347~positive regulation of transferase activity	6.00E-03		
		GO:0043366~beta selection	6.10E-03		
		GO:0044262~cellular carbohydrate metabolic process	6.20E-03		

		GO:0018209~peptidyl-serine modification	6.30E-03		
		GO:0006109~regulation of carbohydrate metabolic process	6.30E-03		
		GO:0060341~regulation of cellular localization	6.70E-03		
		GO:0035239~tube morphogenesis	6.80E-03		
		GO:0010627~regulation of protein kinase cascade	6.80E-03		
		GO:0050714~positive regulation of protein secretion	6.90E-03		
		GO:0002700~regulation of production of molecular mediator of immune response	6.90E-03		
		GO:0043270~positive regulation of ion transport	7.20E-03		
		GO:0032870~cellular response to hormone stimulus	7.70E-03		
		GO:0050801~ion homeostasis	7.80E-03		
		GO:0002252~immune effector process	7.80E-03		
		GO:0048593~camera-type eye morphogenesis	7.90E-03		
		GO:0046890~regulation of lipid biosynthetic process	8.30E-03		
		GO:0042326~negative regulation of phosphorylation	8.30E-03		
		GO:0009416~response to light stimulus	8.50E-03		
		GO:0007005~mitochondrion organization	8.50E-03		
		GO:0048660~regulation of smooth muscle cell proliferation	8.60E-03		
		GO:0045165~cell fate commitment	8.60E-03		
		GO:0009653~anatomical structure morphogenesis	8.90E-03		
		GO:0007417~central nervous system development	9.10E-03		
		GO:0006808~regulation of nitrogen utilization	9.10E-03		
		GO:0048523~negative regulation of cellular process	9.30E-03		
		GO:0010563~negative regulation of phosphorus metabolic process	9.40E-03		
		GO:0030183~B cell differentiation	9.40E-03		
		GO:0045936~negative regulation of phosphate metabolic process	9.40E-03		
		GO:0080134~regulation of	9.50E-03		

		response to stress			
		GO:0040011~locomotion	9.60E-03		
		GO:0016477~cell migration	9.70E-03		
		GO:0050778~positive regulation of immune response	9.70E-03		
		GO:0019217~regulation of fatty acid metabolic process	9.70E-03		
		GO:0009896~positive regulation of catabolic process	9.70E-03		
		GO:0045580~regulation of T cell differentiation	1.10E-02		
		GO:0055080~cation homeostasis	1.10E-02		
		GO:0045935~positive regulation of nucleobase, nucleoside, nucleotide and nucleic acid metabolic process	1.10E-02		
		GO:0010746~regulation of plasma membrane long-chain fatty acid transport	1.20E-02		
		GO:0010748~negative regulation of plasma membrane long-chain fatty acid transport	1.20E-02		
		GO:0043497~regulation of protein heterodimerization activity	1.20E-02		
		GO:0031558~induction of apoptosis in response to chemical stimulus	1.20E-02		
		GO:0009408~response to heat	1.30E-02		
		GO:0046903~secretion	1.30E-02		
		GO:0010565~regulation of cellular ketone metabolic process	1.30E-02		
		GO:0050708~regulation of protein secretion	1.30E-02		
		GO:0007610~behavior	1.30E-02		
		GO:0051241~negative regulation of multicellular organismal process	1.40E-02		
		GO:0051674~localization of cell	1.40E-02		
		GO:0048870~cell motility	1.40E-02		
		GO:0045893~positive regulation of transcription, DNA-dependent	1.40E-02		
		GO:0040014~regulation of multicellular organism growth	1.50E-02		
		GO:0002703~regulation of leukocyte mediated immunity	1.50E-02		
		GO:0031329~regulation of cellular catabolic process	1.50E-02		
		GO:0009636~response to toxin	1.50E-02		
		GO:0051254~positive regulation	1.50E-02		

		of RNA metabolic process			
		GO:0048087~positive regulation of pigmentation during development	1.50E-02		
		GO:0070059~apoptosis in response to endoplasmic reticulum stress	1.50E-02		
		GO:0031557~induction of programmed cell death in response to chemical stimulus	1.50E-02		
		GO:0048609~reproductive process in a multicellular organism	1.60E-02		
		GO:0032504~multicellular organism reproduction	1.60E-02		
		GO:0045619~regulation of lymphocyte differentiation	1.60E-02		
		GO:0031668~cellular response to extracellular stimulus	1.60E-02		
		GO:0006954~inflammatory response	1.70E-02		
		GO:0051222~positive regulation of protein transport	1.80E-02		
		GO:0051726~regulation of cell cycle	1.80E-02		
		GO:0006874~cellular calcium ion homeostasis	1.80E-02		
		GO:0048304~positive regulation of isotype switching to IgG isotypes	1.80E-02		
		GO:0030258~lipid modification	1.90E-02		
		GO:0048592~eye morphogenesis	1.90E-02		
		GO:0070302~regulation of stress-activated protein kinase signaling pathway	1.90E-02		
		GO:0010720~positive regulation of cell development	1.90E-02		
		GO:0007229~integrin-mediated signaling pathway	1.90E-02		
		GO:0050678~regulation of epithelial cell proliferation	2.00E-02		
		GO:0051960~regulation of nervous system development	2.10E-02		
		GO:0050663~cytokine secretion	2.10E-02		
		GO:0010893~positive regulation of steroid biosynthetic process	2.10E-02		
		GO:0043496~regulation of protein homodimerization activity	2.10E-02		
		GO:0046668~regulation of retinal	2.10E-02		

		cell programmed cell death			
		GO:0044255~cellular lipid metabolic process	2.10E-02		
		GO:0006875~cellular metal ion homeostasis	2.20E-02		
		GO:0005975~carbohydrate metabolic process	2.20E-02		
		GO:0048646~anatomical structure formation involved in morphogenesis	2.20E-02		
		GO:0048469~cell maturation	2.20E-02		
		GO:0031667~response to nutrient levels	2.20E-02		
		GO:0009314~response to radiation	2.30E-02		
		GO:0051046~regulation of secretion	2.30E-02		
		GO:0008285~negative regulation of cell proliferation	2.40E-02		
		GO:0034097~response to cytokine stimulus	2.40E-02		
		GO:0002902~regulation of B cell apoptosis	2.40E-02		
		GO:0048302~regulation of isotype switching to IgG isotypes	2.40E-02		
		GO:0031998~regulation of fatty acid beta-oxidation	2.40E-02		
		GO:0032469~endoplasmic reticulum calcium ion homeostasis	2.40E-02		
		GO:0060284~regulation of cell development	2.40E-02		
		GO:0045927~positive regulation of growth	2.50E-02		
		GO:0000187~activation of MAPK activity	2.60E-02		
		GO:0045944~positive regulation of transcription from RNA polymerase II promoter	2.60E-02		
		GO:0009266~response to temperature stimulus	2.60E-02		
		GO:0006873~cellular ion homeostasis	2.70E-02		
		GO:0046641~positive regulation of alpha-beta T cell proliferation	2.70E-02		
		GO:0043368~positive T cell selection	2.70E-02		
		GO:0045830~positive regulation of isotype switching	2.70E-02		
		GO:0002262~myeloid cell	2.70E-02		

		homeostasis			
		GO:0043491~protein kinase B signaling cascade	2.70E-02		
		GO:0045911~positive regulation of DNA recombination	2.70E-02		
		GO:0009887~organ morphogenesis	2.80E-02		
		GO:0045941~positive regulation of transcription	2.80E-02		
		GO:0046777~protein amino acid autophosphorylation	2.80E-02		
		GO:0055082~cellular chemical homeostasis	2.80E-02		
		GO:0006606~protein import into nucleus	2.80E-02		
		GO:0002443~leukocyte mediated immunity	2.80E-02		
		GO:0006469~negative regulation of protein kinase activity	2.90E-02		
		GO:0051170~nuclear import	2.90E-02		
		GO:0070227~lymphocyte apoptosis	3.00E-02		
		GO:0032891~negative regulation of organic acid transport	3.00E-02		
		GO:0048875~chemical homeostasis within a tissue	3.00E-02		
		GO:0043129~surfactant homeostasis	3.00E-02		
		GO:0045725~positive regulation of glycogen biosynthetic process	3.00E-02		
		GO:0033138~positive regulation of peptidyl-serine phosphorylation	3.00E-02		
		GO:0033673~negative regulation of kinase activity	3.10E-02		
		GO:0043523~regulation of neuron apoptosis	3.10E-02		
		GO:0010628~positive regulation of gene expression	3.10E-02		
		GO:0060429~epithelium development	3.20E-02		
		GO:0030005~cellular di-, tri-valent inorganic cation homeostasis	3.20E-02		
		GO:0051881~regulation of mitochondrial membrane potential	3.30E-02		
		GO:0001782~B cell homeostasis	3.30E-02		
		GO:0048070~regulation of pigmentation during development	3.30E-02		
		GO:0046902~regulation of	3.30E-02		

		mitochondrial membrane permeability			
		GO:0034504~protein localization in nucleus	3.30E-02		
		GO:0019932~second-messenger-mediated signaling	3.50E-02		
		GO:0001822~kidney development	3.50E-02		
		GO:0009894~regulation of catabolic process	3.50E-02		
		GO:0051348~negative regulation of transferase activity	3.50E-02		
		GO:0034613~cellular protein localization	3.60E-02		
		GO:0070228~regulation of lymphocyte apoptosis	3.60E-02		
		GO:0030324~lung development	3.60E-02		
		GO:0070727~cellular macromolecule localization	3.70E-02		
		GO:0048585~negative regulation of response to stimulus	3.70E-02		
		GO:0007281~germ cell development	3.80E-02		
		GO:0051240~positive regulation of multicellular organismal process	3.80E-02		
		GO:0030323~respiratory tube development	3.90E-02		
		GO:0043406~positive regulation of MAP kinase activity	3.90E-02		
		GO:0032770~positive regulation of monooxygenase activity	3.90E-02		
		GO:0046640~regulation of alpha-beta T cell proliferation	3.90E-02		
		GO:0045822~negative regulation of heart contraction	3.90E-02		
		GO:0042100~B cell proliferation	3.90E-02		
		GO:0045191~regulation of isotype switching	3.90E-02		
		GO:0006066~alcohol metabolic process	4.10E-02		
		GO:0005979~regulation of glycogen biosynthetic process	4.20E-02		
		GO:0010962~regulation of glucan biosynthetic process	4.20E-02		
		GO:0032885~regulation of polysaccharide biosynthetic process	4.20E-02		
		GO:0045940~positive regulation of steroid metabolic process	4.20E-02		

		GO:0030003~cellular cation homeostasis	4.20E-02		
		GO:0051789~response to protein stimulus	4.20E-02		
		GO:0043010~camera-type eye development	4.20E-02		
		GO:0060541~respiratory system development	4.30E-02		
		GO:0031323~regulation of cellular metabolic process	4.40E-02		
		GO:0001655~urogenital system development	4.40E-02		
		GO:0032369~negative regulation of lipid transport	4.50E-02		
		GO:0032881~regulation of polysaccharide metabolic process	4.50E-02		
		GO:0051279~regulation of release of sequestered calcium ion into cytosol	4.50E-02		
		GO:0006810~transport	4.50E-02		
		GO:0045598~regulation of fat cell differentiation	4.80E-02		
		GO:0006887~exocytosis	4.80E-02		
		GO:0051234~establishment of localization	4.80E-02		
		GO:0046907~intracellular transport	4.80E-02		
		GO:0008104~protein localization	4.90E-02		
$M_{(s4)}$	stat1, smad2, f9, rela, ccl2, osm, myc, timp1, pold3, cdkn2a, igf1, igf1r, csf1, ccl5, tp53, ccl3, csf3, ccl11, sod1	GO:0042127~regulation of cell proliferation	1.10E-11	Pathways in cancer	4.80E-05
		GO:0008284~positive regulation of cell proliferation	5.70E-10	Pancreatic cancer	7.70E-05
		GO:0048522~positive regulation of cellular process	7.50E-10	Cytokine-cytokine receptor interaction	1.40E-04
		GO:0048518~positive regulation of biological process	2.70E-09	Chemokine signaling pathway	2.80E-04
		GO:0002376~immune system process	4.20E-09	NOD-like receptor signaling pathway	1.00E-03
		GO:0065008~regulation of biological quality	1.60E-08	Glioma	1.10E-03
		GO:0009605~response to external stimulus	3.40E-08	Melanoma	1.50E-03
		GO:0042592~homeostatic process	1.00E-07	Chronic myeloid leukemia	1.80E-03
		GO:0009628~response to abiotic stimulus	1.90E-07	Colorectal cancer	2.50E-03
		GO:0050896~response to stimulus	3.00E-07	Prostate cancer	3.00E-03
		GO:0030003~cellular cation homeostasis	5.00E-07	Toll-like receptor signaling pathway	4.20E-03

		GO:0042221~response to chemical stimulus	8.10E-07	Cell cycle	7.70E-03
		GO:0006955~immune response	8.90E-07	Bladder cancer	8.40E-03
		GO:0006950~response to stress	9.70E-07	Jak-STAT signaling pathway	1.40E-02
		GO:0055080~cation homeostasis	9.90E-07	p53 signaling pathway	2.10E-02
		GO:0048513~organ development	1.30E-06	Small cell lung cancer	3.10E-02
		GO:0045595~regulation of cell differentiation	1.40E-06		
		GO:0048878~chemical homeostasis	1.80E-06		
		GO:0048519~negative regulation of biological process	2.00E-06		
		GO:0009611~response to wounding	2.20E-06		
		GO:0048731~system development	2.80E-06		
		GO:0042981~regulation of apoptosis	2.80E-06		
		GO:0043067~regulation of programmed cell death	3.00E-06		
		GO:0010941~regulation of cell death	3.10E-06		
		GO:0009887~organ morphogenesis	3.40E-06		
		GO:0043066~negative regulation of apoptosis	3.40E-06		
		GO:0043069~negative regulation of programmed cell death	3.70E-06		
		GO:0060548~negative regulation of cell death	3.80E-06		
		GO:0006916~anti-apoptosis	4.60E-06		
		GO:0006873~cellular ion homeostasis	4.70E-06		
		GO:0055082~cellular chemical homeostasis	5.20E-06		
		GO:0042493~response to drug	5.80E-06		
		GO:0048856~anatomical structure development	6.70E-06		
		GO:0030005~cellular di-, tri-valent inorganic cation homeostasis	7.40E-06		
		GO:0050801~ion homeostasis	7.90E-06		
		GO:0051239~regulation of multicellular organismal process	8.80E-06		
		GO:0048523~negative regulation of cellular process	8.80E-06		
		GO:0032502~developmental process	9.00E-06		
		GO:0055066~di-, tri-valent inorganic cation homeostasis	9.50E-06		

		GO:0007568~aging	9.80E-06		
		GO:0050793~regulation of developmental process	1.10E-05		
		GO:0019725~cellular homeostasis	1.70E-05		
		GO:0010033~response to organic substance	1.70E-05		
		GO:0007165~signal transduction	2.40E-05		
		GO:0007275~multicellular organismal development	2.60E-05		
		GO:0051716~cellular response to stimulus	3.90E-05		
		GO:0006919~activation of caspase activity	4.10E-05		
		GO:0032501~multicellular organismal process	4.50E-05		
		GO:0009612~response to mechanical stimulus	4.60E-05		
		GO:0010604~positive regulation of macromolecule metabolic process	5.20E-05		
		GO:0009653~anatomical structure morphogenesis	5.30E-05		
		GO:0040008~regulation of growth	5.30E-05		
		GO:0010952~positive regulation of peptidase activity	5.40E-05		
		GO:0043280~positive regulation of caspase activity	5.40E-05		
		GO:0010628~positive regulation of gene expression	5.80E-05		
		GO:0044093~positive regulation of molecular function	6.00E-05		
		GO:0031325~positive regulation of cellular metabolic process	6.20E-05		
		GO:0045944~positive regulation of transcription from RNA polymerase II promoter	7.90E-05		
		GO:0009893~positive regulation of metabolic process	8.30E-05		
		GO:0045935~positive regulation of nucleobase, nucleoside, nucleotide and nucleic acid metabolic process	8.60E-05		
		GO:0019221~cytokine-mediated signaling pathway	9.00E-05		
		GO:0009607~response to biotic stimulus	9.30E-05		
		GO:0031667~response to nutrient levels	9.70E-05		

		GO:0051173~positive regulation of nitrogen compound metabolic process	1.00E-04		
		GO:0009314~response to radiation	1.00E-04		
		GO:0010557~positive regulation of macromolecule biosynthetic process	1.10E-04		
		GO:0065009~regulation of molecular function	1.10E-04		
		GO:0032496~response to lipopolysaccharide	1.20E-04		
		GO:0006468~protein amino acid phosphorylation	1.20E-04		
		GO:0043281~regulation of caspase activity	1.30E-04		
		GO:0051704~multi-organism process	1.40E-04		
		GO:0031328~positive regulation of cellular biosynthetic process	1.40E-04		
		GO:0052548~regulation of endopeptidase activity	1.40E-04		
		GO:0009991~response to extracellular stimulus	1.50E-04		
		GO:0009891~positive regulation of biosynthetic process	1.60E-04		
		GO:0052547~regulation of peptidase activity	1.70E-04		
		GO:0002237~response to molecule of bacterial origin	1.70E-04		
		GO:0065007~biological regulation	1.80E-04		
		GO:0007610~behavior	2.40E-04		
		GO:0045893~positive regulation of transcription, DNA-dependent	2.60E-04		
		GO:0006309~DNA fragmentation involved in apoptosis	2.60E-04		
		GO:0032868~response to insulin stimulus	2.60E-04		
		GO:0051254~positive regulation of RNA metabolic process	2.70E-04		
		GO:0048534~hemopoietic or lymphoid organ development	2.80E-04		
		GO:0009892~negative regulation of metabolic process	2.90E-04		
		GO:0016310~phosphorylation	3.40E-04		
		GO:0009615~response to virus	3.40E-04		
		GO:0006259~DNA metabolic process	3.40E-04		
		GO:0007626~locomotory behavior	3.40E-04		

		GO:0002520~immune system development	3.50E-04		
		GO:0006921~cell structure disassembly during apoptosis	3.80E-04		
		GO:0043085~positive regulation of catalytic activity	3.80E-04		
		GO:0051052~regulation of DNA metabolic process	3.80E-04		
		GO:0050794~regulation of cellular process	4.00E-04		
		GO:0051707~response to other organism	4.30E-04		
		GO:0051246~regulation of protein metabolic process	4.80E-04		
		GO:0030154~cell differentiation	4.80E-04		
		GO:0030262~apoptotic nuclear changes	4.90E-04		
		GO:0000737~DNA catabolic process, endonucleolytic	4.90E-04		
		GO:0045941~positive regulation of transcription	5.60E-04		
		GO:0007242~intracellular signaling cascade	5.70E-04		
		GO:0048869~cellular developmental process	6.40E-04		
		GO:0006954~inflammatory response	6.50E-04		
		GO:0080090~regulation of primary metabolic process	6.60E-04		
		GO:0010647~positive regulation of cell communication	6.80E-04		
		GO:0008637~apoptotic mitochondrial changes	7.00E-04		
		GO:0051726~regulation of cell cycle	7.00E-04		
		GO:0050789~regulation of biological process	7.20E-04		
		GO:0006915~apoptosis	7.50E-04		
		GO:0007569~cell aging	7.90E-04		
		GO:0012501~programmed cell death	8.00E-04		
		GO:0007346~regulation of mitotic cell cycle	8.90E-04		
		GO:0043434~response to peptide hormone stimulus	9.20E-04		
		GO:0006793~phosphorus metabolic process	9.50E-04		
		GO:0006796~phosphate metabolic	9.50E-04		

		process			
		GO:0006935~chemotaxis	1.00E-03		
		GO:0042330~taxis	1.00E-03		
		GO:0009725~response to hormone stimulus	1.00E-03		
		GO:0031323~regulation of cellular metabolic process	1.00E-03		
		GO:0007243~protein kinase cascade	1.10E-03		
		GO:0006979~response to oxidative stress	1.10E-03		
		GO:0007259~JAK-STAT cascade	1.10E-03		
		GO:0002682~regulation of immune system process	1.20E-03		
		GO:0010646~regulation of cell communication	1.30E-03		
		GO:0051345~positive regulation of hydrolase activity	1.40E-03		
		GO:0009719~response to endogenous stimulus	1.50E-03		
		GO:0006874~cellular calcium ion homeostasis	1.50E-03		
		GO:0019222~regulation of metabolic process	1.60E-03		
		GO:0055074~calcium ion homeostasis	1.60E-03		
		GO:0008219~cell death	1.70E-03		
		GO:0016265~death	1.70E-03		
		GO:0006357~regulation of transcription from RNA polymerase II promoter	1.70E-03		
		GO:0009617~response to bacterium	1.80E-03		
		GO:0010605~negative regulation of macromolecule metabolic process	1.80E-03		
		GO:0006875~cellular metal ion homeostasis	1.80E-03		
		GO:0043065~positive regulation of apoptosis	1.90E-03		
		GO:0043068~positive regulation of programmed cell death	1.90E-03		
		GO:0010942~positive regulation of cell death	1.90E-03		
		GO:0008283~cell proliferation	1.90E-03		
		GO:0006997~nucleus organization	2.00E-03		
		GO:0022411~cellular component disassembly	2.00E-03		

		GO:0055065~metal ion homeostasis	2.10E-03		
		GO:0042542~response to hydrogen peroxide	2.30E-03		
		GO:0009408~response to heat	2.30E-03		
		GO:0042325~regulation of phosphorylation	2.50E-03		
		GO:0006308~DNA catabolic process	2.60E-03		
		GO:0043687~post-translational protein modification	2.60E-03		
		GO:0032268~regulation of cellular protein metabolic process	2.60E-03		
		GO:0040014~regulation of multicellular organism growth	2.70E-03		
		GO:0006275~regulation of DNA replication	2.80E-03		
		GO:0019220~regulation of phosphate metabolic process	2.90E-03		
		GO:0051174~regulation of phosphorus metabolic process	2.90E-03		
		GO:0045597~positive regulation of cell differentiation	2.90E-03		
		GO:0060255~regulation of macromolecule metabolic process	2.90E-03		
		GO:0030097~hemopoiesis	3.10E-03		
		GO:0050790~regulation of catalytic activity	3.40E-03		
		GO:0051240~positive regulation of multicellular organismal process	3.40E-03		
		GO:0031349~positive regulation of defense response	3.80E-03		
		GO:0009966~regulation of signal transduction	4.00E-03		
		GO:0000302~response to reactive oxygen species	4.00E-03		
		GO:0019538~protein metabolic process	4.10E-03		
		GO:0006355~regulation of transcription, DNA-dependent	4.40E-03		
		GO:0034097~response to cytokine stimulus	4.50E-03		
		GO:0009266~response to temperature stimulus	4.90E-03		
		GO:0051094~positive regulation of developmental process	5.00E-03		
		GO:0051252~regulation of RNA metabolic process	5.00E-03		

		GO:0043193~positive regulation of gene-specific transcription	5.40E-03		
		GO:0030335~positive regulation of cell migration	5.60E-03		
		GO:0006351~transcription, DNA-dependent	5.70E-03		
		GO:0009967~positive regulation of signal transduction	5.80E-03		
		GO:0032774~RNA biosynthetic process	5.90E-03		
		GO:0009889~regulation of biosynthetic process	6.00E-03		
		GO:0030099~myeloid cell differentiation	6.10E-03		
		GO:0022008~neurogenesis	6.20E-03		
		GO:0043170~macromolecule metabolic process	6.40E-03		
		GO:0032879~regulation of localization	6.50E-03		
		GO:0009894~regulation of catabolic process	6.50E-03		
		GO:0048598~embryonic morphogenesis	6.50E-03		
		GO:0006952~defense response	6.70E-03		
		GO:0040017~positive regulation of locomotion	6.80E-03		
		GO:0051272~positive regulation of cell motion	6.80E-03		
		GO:0048872~homeostasis of number of cells	7.10E-03		
		GO:0006917~induction of apoptosis	7.30E-03		
		GO:0006464~protein modification process	7.30E-03		
		GO:0012502~induction of programmed cell death	7.40E-03		
		GO:0007050~cell cycle arrest	7.50E-03		
		GO:0045672~positive regulation of osteoclast differentiation	7.60E-03		
		GO:0007260~tyrosine phosphorylation of STAT protein	7.60E-03		
		GO:0051336~regulation of hydrolase activity	8.40E-03		
		GO:0048247~lymphocyte chemotaxis	8.90E-03		
		GO:0010564~regulation of cell cycle process	9.10E-03		
		GO:0043412~biopolymer	9.30E-03		

		modification			
		GO:0007399~nervous system development	9.90E-03		
		GO:0044260~cellular macromolecule metabolic process	1.00E-02		
		GO:0008285~negative regulation of cell proliferation	1.00E-02		
		GO:0002521~leukocyte differentiation	1.20E-02		
		GO:0032583~regulation of gene-specific transcription	1.20E-02		
		GO:0007005~mitochondrion organization	1.30E-02		
		GO:0007584~response to nutrient	1.30E-02		
		GO:0033081~regulation of T cell differentiation in the thymus	1.40E-02		
		GO:0031347~regulation of defense response	1.40E-02		
		GO:0002675~positive regulation of acute inflammatory response	1.50E-02		
		GO:0019219~regulation of nucleobase, nucleoside, nucleotide and nucleic acid metabolic process	1.60E-02		
		GO:0051098~regulation of binding	1.60E-02		
		GO:0007154~cell communication	1.60E-02		
		GO:0040011~locomotion	1.60E-02		
		GO:0010556~regulation of macromolecule biosynthetic process	1.60E-02		
		GO:0051171~regulation of nitrogen compound metabolic process	1.70E-02		
		GO:0010468~regulation of gene expression	1.70E-02		
		GO:0045069~regulation of viral genome replication	1.80E-02		
		GO:0010740~positive regulation of protein kinase cascade	1.90E-02		
		GO:0030334~regulation of cell migration	1.90E-02		
		GO:0046579~positive regulation of Ras protein signal transduction	2.00E-02		
		GO:0044267~cellular protein metabolic process	2.10E-02		
		GO:0031326~regulation of cellular biosynthetic process	2.10E-02		
		GO:0006928~cell motion	2.10E-02		
		GO:0051057~positive regulation	2.10E-02		

		of small GTPase mediated signal transduction			
		GO:0043200~response to amino acid stimulus	2.10E-02		
		GO:0042177~negative regulation of protein catabolic process	2.10E-02		
		GO:0002763~positive regulation of myeloid leukocyte differentiation	2.30E-02		
		GO:0009303~rRNA transcription	2.30E-02		
		GO:0051248~negative regulation of protein metabolic process	2.30E-02		
		GO:0040012~regulation of locomotion	2.40E-02		
		GO:0048545~response to steroid hormone stimulus	2.40E-02		
		GO:0051960~regulation of nervous system development	2.40E-02		
		GO:0051270~regulation of cell motion	2.50E-02		
		GO:0006461~protein complex assembly	2.50E-02		
		GO:0070271~protein complex biogenesis	2.50E-02		
		GO:0032570~response to progesterone stimulus	2.50E-02		
		GO:0050792~regulation of viral reproduction	2.60E-02		
		GO:0002673~regulation of acute inflammatory response	2.60E-02		
		GO:0001836~release of cytochrome c from mitochondria	2.60E-02		
		GO:0060284~regulation of cell development	2.80E-02		
		GO:0010035~response to inorganic substance	2.80E-02		
		GO:0045670~regulation of osteoclast differentiation	2.80E-02		
		GO:0016070~RNA metabolic process	2.80E-02		
		GO:0032940~secretion by cell	2.80E-02		
		GO:0010332~response to gamma radiation	2.90E-02		
		GO:0001101~response to acid	3.00E-02		
		GO:0043331~response to dsRNA	3.10E-02		
		GO:0048699~generation of neurons	3.20E-02		
		GO:0009790~embryonic	3.40E-02		

		development			
		GO:0001952~regulation of cell-matrix adhesion	3.40E-02		
		GO:0045449~regulation of transcription	3.40E-02		
		GO:0044085~cellular component biogenesis	3.50E-02		
		GO:0032270~positive regulation of cellular protein metabolic process	3.50E-02		
		GO:0045740~positive regulation of DNA replication	3.50E-02		
		GO:0019932~second-messenger-mediated signaling	3.50E-02		
		GO:0048584~positive regulation of response to stimulus	3.60E-02		
		GO:0009987~cellular process	3.70E-02		
		GO:0050729~positive regulation of inflammatory response	3.80E-02		
		GO:0051247~positive regulation of protein metabolic process	3.80E-02		
		GO:0006879~cellular iron ion homeostasis	3.90E-02		
		GO:0048661~positive regulation of smooth muscle cell proliferation	3.90E-02		
		GO:0010627~regulation of protein kinase cascade	3.90E-02		
		GO:0044238~primary metabolic process	4.00E-02		
		GO:0045639~positive regulation of myeloid cell differentiation	4.10E-02		
		GO:0002573~myeloid leukocyte differentiation	4.30E-02		
		GO:0003006~reproductive developmental process	4.30E-02		
		GO:0048145~regulation of fibroblast proliferation	4.40E-02		
		GO:0055072~iron ion homeostasis	4.50E-02		
		GO:0007422~peripheral nervous system development	4.50E-02		
		GO:0009895~negative regulation of catabolic process	4.60E-02		
		GO:0030595~leukocyte chemotaxis	4.60E-02		
		GO:0048468~cell development	4.60E-02		
		GO:0080134~regulation of response to stress	4.70E-02		
		GO:0006139~nucleobase,	4.70E-02		

		nucleoside, nucleotide and nucleic acid metabolic process			
		GO:0051262~protein tetramerization	4.90E-02		
		GO:0060326~cell chemotaxis	4.90E-02		
		GO:0014075~response to amine stimulus	4.90E-02		
$M_{(s5)}$	slc16a1, slc16a3, slc16a7, slc16a8, cma1, slc16a4	-		-	
$M_{(s6)}$	eng, mcam, itgam, ptprc, cd34	-		Cell adhesion molecules (CAMs)	6.80E-04
				Hematopoietic cell lineage	3.10E-02
$M_{(s7)}$	actb, il1rn, il1r2, pgk1, il1b	-		Hematopoietic cell lineage	4.70E-02
$M_{(s8)}$	cdc25c, ifi27, cdk2, cdkn1a	GO:0051329~interphase of mitotic cell cycle	5.30E-05	-	
		GO:0051325~interphase	5.60E-05		
		GO:0051726~regulation of cell cycle	5.50E-04		
		GO:0000278~mitotic cell cycle	6.90E-04		
		GO:0007089~traversing start control point of mitotic cell cycle	8.50E-04		
		GO:0022403~cell cycle phase	8.60E-04		
		GO:0022402~cell cycle process	1.60E-03		
		GO:0000080~G1 phase of mitotic cell cycle	2.50E-03		
		GO:0051318~G1 phase	3.00E-03		
		GO:0000086~G2/M transition of mitotic cell cycle	3.00E-03		
		GO:0007049~cell cycle	3.00E-03		
		GO:0000079~regulation of cyclin-dependent protein kinase activity	7.60E-03		
		GO:0007346~regulation of mitotic cell cycle	2.10E-02		
		GO:0006260~DNA replication	2.70E-02		
		GO:0007067~mitosis	3.10E-02		
		GO:0000280~nuclear division	3.10E-02		
		GO:0000087~M phase of mitotic cell cycle	3.10E-02		
		GO:0048285~organelle fission	3.20E-02		
		GO:0051301~cell division	4.10E-02		
		GO:0000279~M phase	4.60E-02		

		GO:0045859~regulation of protein kinase activity	4.80E-02		
$M_{(s9)}$	ptgfrn, esr2, gja1, ptger4	GO:0001764~neuron migration	6.60E-03	-	
		GO:0008285~negative regulation of cell proliferation	2.30E-02		
		GO:0016477~cell migration	2.40E-02		
		GO:0048870~cell motility	2.60E-02		
		GO:0051674~localization of cell	2.60E-02		
		GO:0006928~cell motion	3.10E-02		
		GO:0040011~locomotion	3.40E-02		
		GO:0048699~generation of neurons	3.50E-02		
		GO:0022008~neurogenesis	3.90E-02		
$M_{(s10)}$	cd68, snai2, fap, actin	-		-	
$M_{(s11)}$	cxcr4, par1, itk, mirn200b	GO:0006952~defense response	4.40E-02	Leukocyte transendothelial migration	2.30E-02
		GO:0006468~protein amino acid phosphorylation	4.70E-02	Chemokine signaling pathway	3.70E-02

Table 5

1042 GO biological functions and 71 KEGG pathways in top 10 non-overlapping modules of Aspirin network

Modules	Genes	GOTERM_BP_ALL		KEGG_PATHWAY	
		Term	P-value	Term	P-value
$M_{(a1)}$	idh2, ccr6, dlst, il23r, ogdh, mcam, tnfsf10, idh3a, ceacam1, eno1, tnfsf13b, aldoa, cd28, gpi, map3k4, fbp1, ptpn13, taldo1, ctsh, pgk1, ltb, arntl, atf5, furin, itgal, cxcr6, mmrn1, tgfbi, icam1, cd40lg, faslg, tnc, itgb2, itga5, col11a1, col5a1, akt1, adamts8, fn1, ctnnd2, tp53, mmp8, clec3b, cdkn1a, timp1, hmi, birc3, bbc3, bnip1,	GO:0042981~regulation of apoptosis	1.10E-13	Alzheimer's disease	5.00E-08
		GO:0043067~regulation of programmed cell death	1.30E-13	Huntington's disease	1.60E-07
		GO:0010941~regulation of cell death	1.50E-13	Parkinson's disease	2.90E-07
		GO:0043065~positive regulation of apoptosis	8.80E-13	Cytokine-cytokine receptor interaction	2.10E-06
		GO:0043068~positive regulation of programmed cell death	1.00E-12	Oxidative phosphorylation	2.90E-06
		GO:0010942~positive regulation of cell death	1.10E-12	ECM-receptor interaction	6.20E-05
		GO:0006091~generation of precursor metabolites and energy	4.90E-12	Apoptosis	7.70E-05
		GO:0006917~induction of apoptosis	7.70E-11	Citrate cycle (TCA cycle)	4.90E-04
		GO:0012502~induction of programmed cell death	8.10E-11	Viral myocarditis	1.60E-03
		GO:0009605~response to external stimulus	9.50E-11	Focal adhesion	2.80E-03

	birc8, casp9, casp7, cox2, col4a3, cd5, utp11l, znf443, tnfrsf1b, tp73, itga6, tiaf1, tnfrsf25, sst, stk17a, cd44, nr3c1, spp1, tnf, il17a, cebpg, runx1, sparc, ctgf, ndufb10, ndufa5, sdha, ndufs1, adamts1, cox5a, uqcrh, atp5a1, ccl20, cox5b, il22, atp5h, il26, atp5b, rorc	GO:0006915~apoptosis	3.80E-10	Pentose phosphate pathway	3.30E-03
		GO:0012501~programmed cell death	4.90E-10	Small cell lung cancer	3.40E-03
		GO:0009611~response to wounding	2.30E-09	Glycolysis / Gluconeogenesis	5.90E-03
		GO:0008219~cell death	8.10E-09	p53 signaling pathway	9.10E-03
		GO:0016265~death	9.10E-09	Allograft rejection	9.30E-03
		GO:0007155~cell adhesion	3.00E-08	Hematopoietic cell lineage	2.00E-02
		GO:0022610~biological adhesion	3.10E-08	Cell adhesion molecules (CAMs)	2.20E-02
		GO:0048522~positive regulation of cellular process	5.10E-08	Natural killer cell mediated cytotoxicity	2.20E-02
		GO:0006954~inflammatory response	7.70E-08	Amyotrophic lateral sclerosis (ALS)	2.60E-02
		GO:0002376~immune system process	8.40E-08	Pathways in cancer	4.40E-02
		GO:0045333~cellular respiration	1.30E-07		
		GO:0048518~positive regulation of biological process	1.30E-07		
		GO:0015980~energy derivation by oxidation of organic compounds	2.20E-07		
		GO:0007159~leukocyte adhesion	6.60E-07		
		GO:0050896~response to stimulus	1.20E-06		
		GO:0006950~response to stress	1.30E-06		
		GO:0043066~negative regulation of apoptosis	1.50E-06		
		GO:0043069~negative regulation of programmed cell death	1.70E-06		
		GO:0060548~negative regulation of cell death	1.80E-06		
		GO:0006119~oxidative phosphorylation	2.10E-06		
		GO:0006955~immune response	3.50E-06		
		GO:0006952~defense response	4.00E-06		
		GO:0007229~integrin-mediated signaling pathway	4.10E-06		
		GO:0006006~glucose metabolic process	4.10E-06		
		GO:0048856~anatomical	5.80E-06		

		structure development			
		GO:0016337~cell-cell adhesion	6.60E-06		
		GO:0016477~cell migration	6.60E-06		
		GO:0043525~positive regulation of neuron apoptosis	8.60E-06		
		GO:0052548~regulation of endopeptidase activity	1.00E-05		
		GO:0040011~locomotion	1.10E-05		
		GO:0052547~regulation of peptidase activity	1.40E-05		
		GO:0051674~localization of cell	1.70E-05		
		GO:0048870~cell motility	1.70E-05		
		GO:0048519~negative regulation of biological process	1.70E-05		
		GO:0043523~regulation of neuron apoptosis	1.80E-05		
		GO:0019318~hexose metabolic process	2.20E-05		
		GO:0048731~system development	2.60E-05		
		GO:0006007~glucose catabolic process	2.70E-05		
		GO:0006928~cell motion	2.90E-05		
		GO:0008637~apoptotic mitochondrial changes	3.60E-05		
		GO:0006916~anti-apoptosis	3.60E-05		
		GO:0048523~negative regulation of cellular process	3.60E-05		
		GO:0022904~respiratory electron transport chain	4.30E-05		
		GO:0009060~aerobic respiration	5.80E-05		
		GO:0005996~monosaccharide metabolic process	6.10E-05		
		GO:0019320~hexose catabolic process	6.20E-05		
		GO:0046365~monosaccharide catabolic process	7.10E-05		
		GO:0048513~organ development	7.60E-05		
		GO:0043281~regulation of caspase activity	1.20E-04		
		GO:0044262~cellular carbohydrate metabolic process	1.20E-04		

		GO:0046164~alcohol catabolic process	1.30E-04		
		GO:0065008~regulation of biological quality	1.50E-04		
		GO:0044275~cellular carbohydrate catabolic process	1.70E-04		
		GO:0001558~regulation of cell growth	1.80E-04		
		GO:0006096~glycolysis	1.90E-04		
		GO:0007275~multicellular organismal development	2.10E-04		
		GO:0032502~developmental process	2.30E-04		
		GO:0032501~multicellular organismal process	2.30E-04		
		GO:0001836~release of cytochrome c from mitochondria	2.70E-04		
		GO:0042176~regulation of protein catabolic process	2.80E-04		
		GO:0006919~activation of caspase activity	3.20E-04		
		GO:0045792~negative regulation of cell size	3.40E-04		
		GO:0006099~tricarboxylic acid cycle	3.50E-04		
		GO:0046356~acetyl-CoA catabolic process	3.50E-04		
		GO:0005975~carbohydrate metabolic process	3.60E-04		
		GO:0042775~mitochondrial ATP synthesis coupled electron transport	3.70E-04		
		GO:0042773~ATP synthesis coupled electron transport	3.70E-04		
		GO:0009411~response to UV	4.50E-04		
		GO:0043280~positive regulation of caspase activity	4.50E-04		
		GO:0010952~positive regulation of peptidase activity	4.50E-04		
		GO:0009109~coenzyme catabolic process	5.10E-04		
		GO:0016052~carbohydrate catabolic process	5.30E-04		
		GO:0045926~negative regulation of growth	5.50E-04		
		GO:0022900~electron transport chain	6.50E-04		

		GO:0033627~cell adhesion mediated by integrin	7.60E-04		
		GO:0006084~acetyl-CoA metabolic process	8.70E-04		
		GO:0051187~cofactor catabolic process	8.70E-04		
		GO:0042127~regulation of cell proliferation	9.10E-04		
		GO:0048534~hemopoietic or lymphoid organ development	1.00E-03		
		GO:0040008~regulation of growth	1.10E-03		
		GO:0065007~biological regulation	1.10E-03		
		GO:0000060~protein import into nucleus, translocation	1.10E-03		
		GO:0050794~regulation of cellular process	1.20E-03		
		GO:0009987~cellular process	1.30E-03		
		GO:0002520~immune system development	1.50E-03		
		GO:0009895~negative regulation of catabolic process	1.50E-03		
		GO:0030155~regulation of cell adhesion	1.50E-03		
		GO:0008285~negative regulation of cell proliferation	1.60E-03		
		GO:0032891~negative regulation of organic acid transport	1.60E-03		
		GO:0009628~response to abiotic stimulus	1.80E-03		
		GO:0001775~cell activation	1.80E-03		
		GO:0050789~regulation of biological process	2.00E-03		
		GO:0006753~nucleoside phosphate metabolic process	2.00E-03		
		GO:0009117~nucleotide metabolic process	2.00E-03		
		GO:0048583~regulation of response to stimulus	2.10E-03		
		GO:0007160~cell-matrix adhesion	2.10E-03		
		GO:0009653~anatomical structure morphogenesis	2.10E-03		
		GO:0022603~regulation of anatomical structure morphogenesis	2.20E-03		

		GO:0009991~response to extracellular stimulus	2.20E-03		
		GO:0002682~regulation of immune system process	2.40E-03		
		GO:0030308~negative regulation of cell growth	2.40E-03		
		GO:0002286~T cell activation during immune response	2.70E-03		
		GO:0051043~regulation of membrane protein ectodomain proteolysis	2.70E-03		
		GO:0009894~regulation of catabolic process	2.80E-03		
		GO:0031328~positive regulation of cellular biosynthetic process	2.90E-03		
		GO:0031589~cell-substrate adhesion	3.00E-03		
		GO:0042108~positive regulation of cytokine biosynthetic process	3.10E-03		
		GO:0055086~nucleobase, nucleoside and nucleotide metabolic process	3.10E-03		
		GO:0030097~hemopoiesis	3.20E-03		
		GO:0009891~positive regulation of biosynthetic process	3.20E-03		
		GO:0016310~phosphorylation	3.20E-03		
		GO:0080135~regulation of cellular response to stress	3.60E-03		
		GO:0009893~positive regulation of metabolic process	3.70E-03		
		GO:0001568~blood vessel development	3.80E-03		
		GO:0046034~ATP metabolic process	3.80E-03		
		GO:0030162~regulation of proteolysis	4.10E-03		
		GO:0010627~regulation of protein kinase cascade	4.10E-03		
		GO:0001944~vasculature development	4.30E-03		
		GO:0008629~induction of apoptosis by intracellular signals	4.30E-03		
		GO:0006066~alcohol	4.60E-03		

		metabolic process			
		GO:0006000~fructose metabolic process	4.70E-03		
		GO:0032890~regulation of organic acid transport	4.70E-03		
		GO:0008624~induction of apoptosis by extracellular signals	4.80E-03		
		GO:0001817~regulation of cytokine production	4.90E-03		
		GO:0045787~positive regulation of cell cycle	5.00E-03		
		GO:0050900~leukocyte migration	5.00E-03		
		GO:0002285~lymphocyte activation during immune response	5.30E-03		
		GO:0010604~positive regulation of macromolecule metabolic process	5.60E-03		
		GO:0009205~purine ribonucleoside triphosphate metabolic process	5.60E-03		
		GO:0006793~phosphorus metabolic process	5.80E-03		
		GO:0006796~phosphate metabolic process	5.80E-03		
		GO:0009199~ribonucleoside triphosphate metabolic process	5.80E-03		
		GO:0031329~regulation of cellular catabolic process	6.10E-03		
		GO:0042060~wound healing	6.20E-03		
		GO:0010557~positive regulation of macromolecule biosynthetic process	6.30E-03		
		GO:0031330~negative regulation of cellular catabolic process	6.50E-03		
		GO:0080134~regulation of response to stress	6.50E-03		
		GO:0009144~purine nucleoside triphosphate metabolic process	6.50E-03		
		GO:0051128~regulation of cellular component organization	6.70E-03		
		GO:0031325~positive regulation of cellular	6.90E-03		

		metabolic process			
		GO:0045429~positive regulation of nitric oxide biosynthetic process	7.10E-03		
		GO:0009887~organ morphogenesis	7.30E-03		
		GO:0046649~lymphocyte activation	7.30E-03		
		GO:0008633~activation of pro-apoptotic gene products	7.80E-03		
		GO:0007157~heterophilic cell adhesion	7.80E-03		
		GO:0009141~nucleoside triphosphate metabolic process	8.40E-03		
		GO:0002521~leukocyte differentiation	8.40E-03		
		GO:0008361~regulation of cell size	8.50E-03		
		GO:0007044~cell-substrate junction assembly	8.50E-03		
		GO:0007154~cell communication	8.70E-03		
		GO:0009150~purine ribonucleotide metabolic process	1.00E-02		
		GO:0007005~mitochondrion organization	1.00E-02		
		GO:0009416~response to light stimulus	1.00E-02		
		GO:0045840~positive regulation of mitosis	1.00E-02		
		GO:0045862~positive regulation of proteolysis	1.00E-02		
		GO:0051785~positive regulation of nuclear division	1.00E-02		
		GO:0042035~regulation of cytokine biosynthetic process	1.00E-02		
		GO:0051239~regulation of multicellular organismal process	1.10E-02		
		GO:0045428~regulation of nitric oxide biosynthetic process	1.20E-02		
		GO:0045732~positive regulation of protein catabolic process	1.20E-02		
		GO:0002291~T cell activation via T cell receptor contact with	1.20E-02		

		antigen bound to MHC molecule on antigen presenting cell			
		GO:0006959~humoral immune response	1.20E-02		
		GO:0009259~ribonucleotide metabolic process	1.20E-02		
		GO:0050670~regulation of lymphocyte proliferation	1.40E-02		
		GO:0065009~regulation of molecular function	1.40E-02		
		GO:0006732~coenzyme metabolic process	1.40E-02		
		GO:0070663~regulation of leukocyte proliferation	1.50E-02		
		GO:0032944~regulation of mononuclear cell proliferation	1.50E-02		
		GO:0006606~protein import into nucleus	1.60E-02		
		GO:0055114~oxidation reduction	1.60E-02		
		GO:0045321~leukocyte activation	1.60E-02		
		GO:0006970~response to osmotic stress	1.60E-02		
		GO:0051173~positive regulation of nitrogen compound metabolic process	1.60E-02		
		GO:0051170~nuclear import	1.70E-02		
		GO:0051336~regulation of hydrolase activity	1.70E-02		
		GO:0006754~ATP biosynthetic process	1.70E-02		
		GO:0046324~regulation of glucose import	1.70E-02		
		GO:0008630~DNA damage response, signal transduction resulting in induction of apoptosis	1.70E-02		
		GO:0051246~regulation of protein metabolic process	1.80E-02		
		GO:0010827~regulation of glucose transport	1.80E-02		
		GO:0043648~dicarboxylic acid metabolic process	1.80E-02		
		GO:0060553~induction of necroptosis	1.80E-02		
		GO:0060545~positive	1.80E-02		

		regulation of necroptosis			
		GO:0060544~regulation of necroptosis	1.80E-02		
		GO:0060555~induction of necroptosis by extracellular signals	1.80E-02		
		GO:0030154~cell differentiation	1.90E-02		
		GO:0030099~myeloid cell differentiation	1.90E-02		
		GO:0034504~protein localization in nucleus	2.00E-02		
		GO:0002263~cell activation during immune response	2.00E-02		
		GO:0031331~positive regulation of cellular catabolic process	2.00E-02		
		GO:0002366~leukocyte activation during immune response	2.00E-02		
		GO:0050793~regulation of developmental process	2.10E-02		
		GO:0051251~positive regulation of lymphocyte activation	2.10E-02		
		GO:0043123~positive regulation of I-kappaB kinase/NF-kappaB cascade	2.10E-02		
		GO:0009206~purine ribonucleoside triphosphate biosynthetic process	2.20E-02		
		GO:0009145~purine nucleoside triphosphate biosynthetic process	2.30E-02		
		GO:0009201~ribonucleoside triphosphate biosynthetic process	2.30E-02		
		GO:0048872~homeostasis of number of cells	2.30E-02		
		GO:0002697~regulation of immune effector process	2.40E-02		
		GO:0051345~positive regulation of hydrolase activity	2.40E-02		
		GO:0002037~negative regulation of L-glutamate transport	2.40E-02		
		GO:0010940~positive regulation of necrotic cell	2.40E-02		

		death			
		GO:0048305~immunoglobulin secretion	2.40E-02		
		GO:0009142~nucleoside triphosphate biosynthetic process	2.40E-02		
		GO:0006769~nicotinamide metabolic process	2.50E-02		
		GO:0046496~nicotinamide nucleotide metabolic process	2.50E-02		
		GO:0015985~energy coupled proton transport, down electrochemical gradient	2.50E-02		
		GO:0015986~ATP synthesis coupled proton transport	2.50E-02		
		GO:0032535~regulation of cellular component size	2.50E-02		
		GO:0030098~lymphocyte differentiation	2.50E-02		
		GO:0032268~regulation of cellular protein metabolic process	2.50E-02		
		GO:0009820~alkaloid metabolic process	2.60E-02		
		GO:0034329~cell junction assembly	2.60E-02		
		GO:0044093~positive regulation of molecular function	2.60E-02		
		GO:0006163~purine nucleotide metabolic process	2.70E-02		
		GO:0002696~positive regulation of leukocyte activation	2.70E-02		
		GO:0019362~pyridine nucleotide metabolic process	2.70E-02		
		GO:0006120~mitochondrial electron transport, NADH to ubiquinone	2.70E-02		
		GO:0048869~cellular developmental process	2.70E-02		
		GO:0051716~cellular response to stimulus	2.70E-02		
		GO:0043122~regulation of I-kappaB kinase/NF-kappaB cascade	2.80E-02		
		GO:0019058~viral infectious cycle	2.80E-02		

		GO:0007267~cell-cell signaling	3.00E-02		
		GO:0009180~purine ribonucleoside diphosphate biosynthetic process	3.00E-02		
		GO:0006102~isocitrate metabolic process	3.00E-02		
		GO:0006172~ADP biosynthetic process	3.00E-02		
		GO:0009136~purine nucleoside diphosphate biosynthetic process	3.00E-02		
		GO:0002347~response to tumor cell	3.00E-02		
		GO:0010939~regulation of necrotic cell death	3.00E-02		
		GO:0002036~regulation of L-glutamate transport	3.00E-02		
		GO:0009188~ribonucleoside diphosphate biosynthetic process	3.00E-02		
		GO:0070265~necrotic cell death	3.00E-02		
		GO:0050867~positive regulation of cell activation	3.00E-02		
		GO:0050768~negative regulation of neurogenesis	3.10E-02		
		GO:0046890~regulation of lipid biosynthetic process	3.10E-02		
		GO:0051179~localization	3.10E-02		
		GO:0010033~response to organic substance	3.10E-02		
		GO:0051186~cofactor metabolic process	3.10E-02		
		GO:0031667~response to nutrient levels	3.20E-02		
		GO:0050790~regulation of catalytic activity	3.30E-02		
		GO:0001503~ossification	3.30E-02		
		GO:0009314~response to radiation	3.40E-02		
		GO:0010721~negative regulation of cell development	3.50E-02		
		GO:0030183~B cell differentiation	3.50E-02		
		GO:0009152~purine ribonucleotide biosynthetic process	3.50E-02		

		GO:0009896~positive regulation of catabolic process	3.60E-02		
		GO:0034220~ion transmembrane transport	3.60E-02		
		GO:0046031~ADP metabolic process	3.60E-02		
		GO:0051956~negative regulation of amino acid transport	3.60E-02		
		GO:0044070~regulation of anion transport	3.60E-02		
		GO:0070201~regulation of establishment of protein localization	3.80E-02		
		GO:0060348~bone development	3.90E-02		
		GO:0006733~oxidoreduction coenzyme metabolic process	4.00E-02		
		GO:0009260~ribonucleotide biosynthetic process	4.00E-02		
		GO:0042110~T cell activation	4.20E-02		
		GO:0009133~nucleoside diphosphate biosynthetic process	4.20E-02		
		GO:0006002~fructose 6-phosphate metabolic process	4.20E-02		
		GO:0008360~regulation of cell shape	4.30E-02		
		GO:0050671~positive regulation of lymphocyte proliferation	4.40E-02		
		GO:0001501~skeletal system development	4.50E-02		
		GO:0032946~positive regulation of mononuclear cell proliferation	4.60E-02		
		GO:0051783~regulation of nuclear division	4.60E-02		
		GO:0009612~response to mechanical stimulus	4.60E-02		
		GO:0007088~regulation of mitosis	4.60E-02		
		GO:0070665~positive regulation of leukocyte proliferation	4.60E-02		
		GO:0043603~cellular amide metabolic process	4.60E-02		
		GO:0017038~protein import	4.60E-02		

		GO:0022604~regulation of cell morphogenesis	4.60E-02		
		GO:0042221~response to chemical stimulus	4.60E-02		
		GO:0034330~cell junction organization	4.70E-02		
		GO:0031294~lymphocyte costimulation	4.80E-02		
		GO:0031295~T cell costimulation	4.80E-02		
		GO:0035313~wound healing, spreading of epidermal cells	4.80E-02		
		GO:0009179~purine ribonucleoside diphosphate metabolic process	4.80E-02		
		GO:0009135~purine nucleoside diphosphate metabolic process	4.80E-02		
		GO:0051953~negative regulation of amine transport	4.80E-02		
		GO:0006734~NADH metabolic process	4.80E-02		
		GO:0002252~immune effector process	4.80E-02		
		GO:0050776~regulation of immune response	4.90E-02		
$M_{(a2)}$	hdac1, dnmt1, ep300, hdac3, mecp2, suv39h1, dnmt3b, jmjd2a, cbx5, sin3a, hdac2, rcor1, ezh2, jmjd3, mbd1, aof2, ptger4, ptger2, dnmt3a	GO:0016568~chromatin modification	1.70E-10	Cysteine and methionine metabolism	1.40E-03
		GO:0006325~chromatin organization	2.20E-10	Huntington's disease	2.90E-03
		GO:0016569~covalent chromatin modification	7.60E-10	Notch signaling pathway	7.40E-02
		GO:0051276~chromosome organization	9.00E-10		
		GO:0006996~organelle organization	4.30E-07		
		GO:0043414~biopolymer methylation	4.20E-06		
		GO:0045892~negative regulation of transcription, DNA-dependent	4.90E-06		
		GO:0051253~negative regulation of RNA metabolic process	5.10E-06		
		GO:0032259~methylation	7.10E-06		
		GO:0050794~regulation of cellular process	8.80E-06		
		GO:0016043~cellular	1.40E-05		

		component organization			
		GO:0016570~histone modification	1.40E-05		
		GO:0016481~negative regulation of transcription	1.40E-05		
		GO:0050789~regulation of biological process	1.60E-05		
		GO:0045934~negative regulation of nucleobase, nucleoside, nucleotide and nucleic acid metabolic process	1.80E-05		
		GO:0051172~negative regulation of nitrogen compound metabolic process	1.90E-05		
		GO:0010629~negative regulation of gene expression	2.00E-05		
		GO:0006305~DNA alkylation	2.10E-05		
		GO:0006306~DNA methylation	2.10E-05		
		GO:0010558~negative regulation of macromolecule biosynthetic process	2.80E-05		
		GO:0016575~histone deacetylation	2.80E-05		
		GO:0006304~DNA modification	2.80E-05		
		GO:0031327~negative regulation of cellular biosynthetic process	3.00E-05		
		GO:0006730~one-carbon metabolic process	3.10E-05		
		GO:0065007~biological regulation	3.30E-05		
		GO:0009890~negative regulation of biosynthetic process	3.40E-05		
		GO:0045449~regulation of transcription	3.70E-05		
$M_{(a3)}$	hspb1, pcsk1, csf1r, socs3, irf6, mmp9, vcam1, gstp1, flt3, lta, crp, lbp, il10, slc10a1, slco2b1, slc22a7, abcb11, abcc2, abcc3, bcr, abcc4,	GO:0042221~response to chemical stimulus	3.10E-14	Metabolism of xenobiotics by cytochrome P450	2.10E-10
		GO:0002237~response to molecule of bacterial origin	2.20E-12	Drug metabolism	6.70E-09
		GO:0032496~response to lipopolysaccharide	2.00E-11	Cytokine-cytokine receptor interaction	4.20E-05
		GO:0010033~response to organic substance	7.70E-11	Jak-STAT signaling pathway	1.30E-04
		GO:0009605~response to external stimulus	1.20E-09	Pathways in cancer	3.10E-04

	slco1b1, slco1b3, abcg2, slc22a1, cyp2b6, cyp2d6, uqcrfs1, cyp1a2, cyp2e1, cyp2c19, cyp2c9, fos, ptk2b, jak2, mmp13, tnfa, gc, adam33, timp2, timp3, serpina1, serpina3, mmp1, crlf2, mmp3, ntrk3, ccl7, tyk2, il7r, cyp19a1, sh2b3, mmp2, abl2, cyp1b1, pdgfrb, epor, elf4, il4, nat2, jun, gstm1, gstm3, gsto1, pon1, pon2, avp, ccl2, cxcl10, mmp12, il6, cyp1a1, abl1, tslp, tnfaip3, nfkbia, egr1, enpp2, hk2, mmp10, gadd45a, wisp1, cebpb, selplg, bcl2a1, vegfa	GO:0009617~response to bacterium	1.40E-08	ABC transporters	9.10E-04
		GO:0017144~drug metabolic process	2.40E-08	Retinol metabolism	2.00E-03
		GO:0030574~collagen catabolic process	8.50E-08	Linoleic acid metabolism	2.60E-03
		GO:0048518~positive regulation of biological process	1.30E-07	Bladder cancer	8.30E-03
		GO:0002376~immune system process	1.60E-07	Hematopoietic cell lineage	1.00E-02
		GO:0048522~positive regulation of cellular process	2.20E-07	Glutathione metabolism	1.30E-02
		GO:0048513~organ development	2.30E-07	Arachidonic acid metabolism	1.80E-02
		GO:0044243~multicellular organismal catabolic process	3.50E-07		
		GO:0014070~response to organic cyclic substance	4.80E-07		
		GO:0032963~collagen metabolic process	5.20E-07		
		GO:0009719~response to endogenous stimulus	5.50E-07		
		GO:0009612~response to mechanical stimulus	8.20E-07		
		GO:0048731~system development	8.60E-07		
		GO:0051707~response to other organism	8.70E-07		
		GO:0044259~multicellular organismal macromolecule metabolic process	8.80E-07		
		GO:0009725~response to hormone stimulus	1.30E-06		
		GO:0048545~response to steroid hormone stimulus	1.60E-06		
		GO:0009611~response to wounding	2.00E-06		
		GO:0009607~response to biotic stimulus	2.10E-06		
		GO:0042127~regulation of cell proliferation	2.20E-06		
		GO:0044236~multicellular organismal metabolic process	2.20E-06		
		GO:0048534~hemopoietic or lymphoid organ development	2.50E-06		
		GO:0042981~regulation of apoptosis	2.90E-06		

		GO:0006916~anti-apoptosis	2.90E-06		
		GO:0006953~acute-phase response	3.30E-06		
		GO:0043067~regulation of programmed cell death	3.30E-06		
		GO:0010941~regulation of cell death	3.50E-06		
		GO:0002520~immune system development	4.30E-06		
		GO:0042493~response to drug	4.30E-06		
		GO:0008284~positive regulation of cell proliferation	4.50E-06		
		GO:0048856~anatomical structure development	5.30E-06		
		GO:0006950~response to stress	5.90E-06		
		GO:0043066~negative regulation of apoptosis	6.00E-06		
		GO:0050896~response to stimulus	6.40E-06		
		GO:0018108~peptidyl-tyrosine phosphorylation	6.60E-06		
		GO:0043069~negative regulation of programmed cell death	6.90E-06		
		GO:0060548~negative regulation of cell death	7.10E-06		
		GO:0051704~multi-organism process	7.50E-06		
		GO:0018212~peptidyl-tyrosine modification	8.20E-06		
		GO:0030097~hemopoiesis	8.90E-06		
		GO:0032879~regulation of localization	9.90E-06		
		GO:0001666~response to hypoxia	1.20E-05		
		GO:0030335~positive regulation of cell migration	1.30E-05		
		GO:0070482~response to oxygen levels	1.70E-05		
		GO:0006954~inflammatory response	1.80E-05		
		GO:0048519~negative regulation of biological process	2.10E-05		
		GO:0051239~regulation of multicellular organismal process	2.20E-05		

		GO:0040017~positive regulation of locomotion	2.20E-05		
		GO:0051272~positive regulation of cell motion	2.20E-05		
		GO:0007275~multicellular organismal development	2.70E-05		
		GO:0006935~chemotaxis	3.90E-05		
		GO:0042330~taxis	3.90E-05		
		GO:0001775~cell activation	4.20E-05		
		GO:0030334~regulation of cell migration	5.60E-05		
		GO:0050865~regulation of cell activation	6.90E-05		
		GO:0048583~regulation of response to stimulus	7.50E-05		
		GO:0002682~regulation of immune system process	7.50E-05		
		GO:0007610~behavior	8.10E-05		
		GO:0032268~regulation of cellular protein metabolic process	8.90E-05		
		GO:0034097~response to cytokine stimulus	9.40E-05		
		GO:0002521~leukocyte differentiation	1.10E-04		
		GO:0040012~regulation of locomotion	1.20E-04		
		GO:0050793~regulation of developmental process	1.30E-04		
		GO:0051270~regulation of cell motion	1.30E-04		
		GO:0048523~negative regulation of cellular process	1.40E-04		
		GO:0031328~positive regulation of cellular biosynthetic process	1.50E-04		
		GO:0007167~enzyme linked receptor protein signaling pathway	1.60E-04		
		GO:0009891~positive regulation of biosynthetic process	1.70E-04		
		GO:0007626~locomotory behavior	1.90E-04		
		GO:0040011~locomotion	1.90E-04		
		GO:0032502~developmental process	1.90E-04		
		GO:0002526~acute	2.60E-04		

		inflammatory response			
		GO:0009628~response to abiotic stimulus	2.80E-04		
		GO:0009991~response to extracellular stimulus	2.90E-04		
		GO:0051246~regulation of protein metabolic process	3.00E-04		
		GO:0051050~positive regulation of transport	3.10E-04		
		GO:0007169~transmembrane receptor protein tyrosine kinase signaling pathway	3.20E-04		
		GO:0051173~positive regulation of nitrogen compound metabolic process	3.20E-04		
		GO:0030098~lymphocyte differentiation	3.30E-04		
		GO:0009404~toxin metabolic process	3.30E-04		
		GO:0050900~leukocyte migration	3.30E-04		
		GO:0032501~multicellular organismal process	3.40E-04		
		GO:0002694~regulation of leukocyte activation	4.00E-04		
		GO:0046677~response to antibiotic	4.50E-04		
		GO:0002684~positive regulation of immune system process	4.60E-04		
		GO:0050867~positive regulation of cell activation	4.60E-04		
		GO:0001932~regulation of protein amino acid phosphorylation	5.00E-04		
		GO:0045321~leukocyte activation	5.10E-04		
		GO:0032103~positive regulation of response to external stimulus	5.20E-04		
		GO:0051223~regulation of protein transport	5.20E-04		
		GO:0045595~regulation of cell differentiation	5.40E-04		
		GO:0006955~immune response	6.00E-04		
		GO:0051222~positive regulation of protein transport	6.10E-04		

		GO:0065008~regulation of biological quality	6.20E-04		
		GO:0070201~regulation of establishment of protein localization	6.90E-04		
		GO:0009893~positive regulation of metabolic process	7.80E-04		
		GO:0006952~defense response	8.20E-04		
		GO:0045935~positive regulation of nucleobase, nucleoside, nucleotide and nucleic acid metabolic process	9.30E-04		
		GO:0031667~response to nutrient levels	9.90E-04		
		GO:0046649~lymphocyte activation	1.00E-03		
		GO:0051099~positive regulation of binding	1.10E-03		
		GO:0051384~response toGlucocorticoid stimulus	1.10E-03		
		GO:0032675~regulation of interleukin-6 production	1.20E-03		
		GO:0032880~regulation of protein localization	1.20E-03		
		GO:0010557~positive regulation of macromolecule biosynthetic process	1.40E-03		
		GO:0045944~positive regulation of transcription from RNA polymerase II promoter	1.40E-03		
		GO:0007259~JAK-STAT cascade	1.50E-03		
		GO:0060326~cell chemotaxis	1.50E-03		
		GO:0031960~response to corticosteroid stimulus	1.50E-03		
		GO:0045941~positive regulation of transcription	1.50E-03		
		GO:0031325~positive regulation of cellular metabolic process	1.60E-03		
		GO:0051249~regulation of lymphocyte activation	1.70E-03		
		GO:0006928~cell motion	1.70E-03		
		GO:0045893~positive regulation of transcription,	1.80E-03		

		DNA-dependent			
		GO:0051254~positive regulation of RNA metabolic process	1.90E-03		
		GO:0010628~positive regulation ofGene expression	1.90E-03		
		GO:0051098~regulation of binding	2.00E-03		
		GO:0050776~regulation of immune response	2.00E-03		
		GO:0015711~organic anion transport	2.10E-03		
		GO:0030154~cell differentiation	2.20E-03		
		GO:0032101~regulation of response to external stimulus	2.30E-03		
		GO:0010646~regulation of cell communication	2.40E-03		
		GO:0048584~positive regulation of response to stimulus	2.50E-03		
		GO:0018193~peptidyl-amino acid modification	2.50E-03		
		GO:0051241~negative regulation of multicellular organismal process	2.60E-03		
		GO:0002697~regulation of immune effector process	2.80E-03		
		GO:0051090~regulation of transcription factor activity	3.00E-03		
		GO:0050864~regulation of B cell activation	3.20E-03		
		GO:0043627~response to estrogen stimulus	3.20E-03		
		GO:0010627~regulation of protein kinase cascade	3.20E-03		
		GO:0002696~positive regulation of leukocyte activation	3.40E-03		
		GO:0048869~cellular developmental process	3.60E-03		
		GO:0051047~positive regulation of secretion	3.70E-03		
		GO:0010604~positive regulation of macromolecule metabolic process	3.80E-03		
		GO:0042592~homeostatic process	4.00E-03		

		GO:0006874~cellular calcium ion homeostasis	4.20E-03		
		GO:0043200~response to amino acid stimulus	4.30E-03		
		GO:0055074~calcium ion homeostasis	4.70E-03		
		GO:0050863~regulation of T cell activation	4.80E-03		
		GO:0008285~negative regulation of cell proliferation	5.00E-03		
		GO:0002684~positive regulation of immune system process	2.00E-02		
		GO:0050867~positive regulation of cell activation	2.00E-02		
		GO:0001932~regulation of protein amino acid phosphorylation	2.00E-02		
		GO:0045321~leukocyte activation	2.00E-02		
		GO:0032103~positive regulation of response to external stimulus	2.00E-02		
		GO:0051223~regulation of protein transport	2.00E-02		
		GO:0045595~regulation of cell differentiation	2.10E-02		
		GO:0006955~immune response	2.20E-02		
		GO:0051222~positive regulation of protein transport	2.20E-02		
		GO:0065008~regulation of biological quality	2.20E-02		
		GO:0070201~regulation of establishment of protein localization	2.30E-02		
		GO:0009893~positive regulation of metabolic process	2.40E-02		
		GO:0006952~defense response	2.50E-02		
		GO:0045935~positive regulation of nucleobase, nucleoside, nucleotide and nucleic acid metabolic process	2.50E-02		
		GO:0031667~response to nutrient levels	2.50E-02		
		GO:0046649~lymphocyte	2.60E-02		

		activation			
		GO:0051099~positive regulation of binding	2.60E-02		
		GO:0051384~response toGlucocorticoid stimulus	2.70E-02		
		GO:0032675~regulation of interleukin-6 production	2.70E-02		
		GO:0032880~regulation of protein localization	2.70E-02		
		GO:0010557~positive regulation of macromolecule biosynthetic process	2.70E-02		
		GO:0045944~positive regulation of transcription from RNA polymerase II promoter	2.70E-02		
		GO:0007259~JAK-STAT cascade	2.80E-02		
		GO:0060326~cell chemotaxis	3.00E-02		
		GO:0031960~response to corticosteroid stimulus	3.00E-02		
		GO:0045941~positive regulation of transcription	3.00E-02		
		GO:0031325~positive regulation of cellular metabolic process	3.00E-02		
		GO:0051249~regulation of lymphocyte activation	3.10E-02		
		GO:0006928~cell motion	3.10E-02		
		GO:0045893~positive regulation of transcription, DNA-dependent	3.20E-02		
		GO:0051254~positive regulation of RNA metabolic process	3.20E-02		
		GO:0010628~positive regulation ofGene expression	3.20E-02		
		GO:0051098~regulation of binding	3.20E-02		
		GO:0050776~regulation of immune response	3.20E-02		
		GO:0015711~organic anion transport	3.20E-02		
		GO:0030154~cell differentiation	3.30E-02		
		GO:0032101~regulation of response to external stimulus	3.30E-02		
		GO:0010646~regulation of	3.30E-02		

		cell communication			
		GO:0048584~positive regulation of response to stimulus	3.40E-02		
		GO:0018193~peptidyl-amino acid modification	3.40E-02		
		GO:0051241~negative regulation of multicellular organismal process	3.50E-02		
		GO:0002697~regulation of immune effector process	3.50E-02		
		GO:0051090~regulation of transcription factor activity	3.50E-02		
		GO:0050864~regulation of B cell activation	3.50E-02		
		GO:0043627~response to estrogen stimulus	3.70E-02		
		GO:0010627~regulation of protein kinase cascade	3.70E-02		
		GO:0002696~positive regulation of leukocyte activation	3.70E-02		
		GO:0048869~cellular developmental process	3.80E-02		
		GO:0051047~positive regulation of secretion	3.80E-02		
		GO:0010604~positive regulation of macromolecule metabolic process	3.80E-02		
		GO:0042592~homeostatic process	3.80E-02		
		GO:0006874~cellular calcium ion homeostasis	3.80E-02		
		GO:0043200~response to amino acid stimulus	3.90E-02		
		GO:0055074~calcium ion homeostasis	3.90E-02		
		GO:0045346~regulation of MHC class II biosynthetic process	4.00E-02		
		GO:0042359~vitamin D metabolic process	4.00E-02		
		GO:0006873~cellular ion homeostasis	4.00E-02		
		GO:0001894~tissue homeostasis	4.00E-02		
		GO:0045471~response to ethanol	4.10E-02		

		GO:0051098~regulation of binding	4.20E-02		
		GO:0055082~cellular chemical homeostasis	4.20E-02		
		GO:0030217~T cell differentiation	4.20E-02		
		GO:0033273~response to vitamin	4.40E-02		
		GO:0048878~chemical homeostasis	4.40E-02		
		GO:0032094~response to food	4.40E-02		
		GO:0035162~embryonic hemopoiesis	4.40E-02		
		GO:0051222~positive regulation of protein transport	4.50E-02		
		GO:0006800~oxygen and reactive oxygen species metabolic process	4.50E-02		
		GO:0007399~nervous system development	4.50E-02		
		GO:0050730~regulation of peptidyl-tyrosine phosphorylation	4.60E-02		
		GO:0042330~taxis	4.70E-02		
		GO:0006935~chemotaxis	4.70E-02		
		GO:0043388~positive regulation of DNA binding	4.80E-02		
		GO:0080134~regulation of response to stress	4.90E-02		
		GO:0007626~locomotory behavior	4.90E-02		
		GO:0009888~tissue development	4.90E-02		
		GO:0042523~positive regulation of tyrosine phosphorylation of Stat5 protein	4.90E-02		
		GO:0048535~lymph node development	4.90E-02		
		GO:0015721~bile acid and bile salt transport	4.90E-02		
$M_{(a4)}$	ptk2, enpp2, pik3ca, mmp13, mapk8, mapk14, cxcl10, gpx4, gpx1, sepn1, ephb2, sepw1, ccl7, elf4,	GO:0042221~response to chemical stimulus	1.90E-15	NOD-like receptor signaling pathway	4.20E-07
		GO:0040011~locomotion	1.40E-13	Toll-like receptor signaling pathway	4.90E-06
		GO:0006928~cell motion	1.50E-12	RIG-I-like receptor signaling pathway	8.50E-04
		GO:0032494~response to	4.10E-12	Chemokine signaling pathway	1.30E-03

	pcsk1, il6, vcam1, nfkbia, uqcrfs1, tnfaip3	peptidoglycan			
		GO:0042330~taxis	4.80E-12	Leukocyte transendothelial migration	3.70E-03
		GO:0006935~chemotaxis	7.40E-12	Neurotrophin signaling pathway	4.20E-03
		GO:0043066~negative regulation of apoptosis	1.10E-11	Cytosolic DNA-sensing pathway	9.60E-03
		GO:0043069~negative regulation of programmed cell death	4.20E-09	Pathways in cancer	1.00E-02
		GO:0060548~negative regulation of cell death	5.00E-09	Epithelial cell signaling in Helicobacter pylori infection	1.40E-02
		GO:0009605~response to external stimulus	5.10E-09	VEGF signaling pathway	1.70E-02
		GO:0009617~response to bacterium	5.40E-09	Fc epsilon RI signaling pathway	1.90E-02
		GO:0002237~response to molecule of bacterial origin	7.00E-09	Small cell lung cancer	2.20E-02
		GO:0048522~positive regulation of cellular process	8.90E-09	Progesterone-mediated oocyte maturation	2.30E-02
		GO:0010033~response to organic substance	1.50E-08	ErbB signaling pathway	2.30E-02
		GO:0050896~response to stimulus	1.60E-08	T cell receptor signaling pathway	3.40E-02
		GO:0007610~behavior	1.80E-08		
		GO:0007626~locomotory behavior	3.60E-08		
		GO:0048518~positive regulation of biological process	4.30E-08		
		GO:0051707~response to other organism	4.70E-08		
		GO:0006950~response to stress	4.80E-08		
		GO:0050789~regulation of biological process	5.50E-08		
		GO:0048513~organ development	7.00E-08		
		GO:0031347~regulation of defense response	8.00E-08		
		GO:0051239~regulation of multicellular organismal process	8.80E-08		
		GO:0007275~multicellular organismal development	8.80E-08		
		GO:0009725~response to hormone stimulus	8.80E-08		
		GO:0048731~system	9.30E-08		

		development			
		GO:0002376~immune system process	1.30E-07		
		GO:0009607~response to biotic stimulus	1.60E-07		
		GO:0002682~regulation of immune system process	2.00E-07		
		GO:0050793~regulation of developmental process	2.70E-07		
		GO:0051704~multi-organism process	2.70E-07		
		GO:0065008~regulation of biological quality	2.80E-07		
		GO:0009719~response to endogenous stimulus	3.60E-07		
		GO:0065007~biological regulation	4.20E-07		
		GO:0048545~response to steroid hormone stimulus	5.40E-07		
		GO:0048856~anatomical structure development	6.00E-07		
		GO:0006518~peptide metabolic process	9.60E-07		
		GO:0010035~response to inorganic substance	9.70E-07		
		GO:0006916~anti-apoptosis	1.30E-06		
		GO:0032502~developmental process	1.50E-06		
		GO:0050794~regulation of cellular process	2.20E-06		
		GO:0042981~regulation of apoptosis	2.50E-06		
		GO:0043067~regulation of programmed cell death	3.20E-06		
		GO:0010941~regulation of cell death	3.50E-06		
		GO:0048523~negative regulation of cellular process	4.90E-06		
		GO:0045595~regulation of cell differentiation	5.60E-06		
		GO:0009611~response to wounding	6.60E-06		
		GO:0050727~regulation of inflammatory response	8.10E-06		
		GO:0032496~response to lipopolysaccharide	8.10E-06		
		GO:0080134~regulation of response to stress	9.40E-06		

		GO:0016477~cell migration	9.40E-06		
		GO:0048519~negative regulation of biological process	1.20E-05		
		GO:0032495~response to muramyl dipeptide	1.30E-05		
		GO:0007267~cell-cell signaling	1.40E-05		
		GO:0006793~phosphorus metabolic process	1.80E-05		
		GO:0006796~phosphate metabolic process	1.80E-05		
		GO:0006915~apoptosis	2.50E-05		
		GO:0032879~regulation of localization	2.80E-05		
		GO:0051674~localization of cell	3.10E-05		
		GO:0048870~cell motility	3.50E-05		
		GO:0012501~programmed cell death	3.60E-05		
		GO:0006468~protein amino acid phosphorylation	3.70E-05		
		GO:0051223~regulation of protein transport	4.10E-05		
		GO:0070201~regulation of establishment of protein localization	4.50E-05		
		GO:0007243~protein kinase cascade	4.60E-05		
		GO:0045944~positive regulation of transcription from RNA polymerase II promoter	4.70E-05		
		GO:0010038~response to metal ion	4.90E-05		
		GO:0008219~cell death	5.30E-05		
		GO:0016265~death	5.90E-05		
		GO:0030154~cell differentiation	6.20E-05		
		GO:0032880~regulation of protein localization	6.50E-05		
		GO:0043687~post-translational protein modification	8.00E-05		
		GO:0031663~lipopolysaccharide-mediated signaling pathway	8.30E-05		
		GO:0001525~angiogenesis	8.80E-05		
		GO:0042127~regulation of cell proliferation	9.20E-05		

		GO:0048869~cellular developmental process	1.10E-04		
		GO:0019538~protein metabolic process	1.20E-04		
		GO:0007154~cell communication	1.50E-04		
		GO:0016310~phosphorylation	1.50E-04		
		GO:0032101~regulation of response to external stimulus	1.60E-04		
		GO:0006629~lipid metabolic process	1.70E-04		
		GO:0051716~cellular response to stimulus	1.80E-04		
		GO:0021955~central nervous system neuron axonogenesis	1.90E-04		
		GO:0030334~regulation of cell migration	2.00E-04		
		GO:0048583~regulation of response to stimulus	2.10E-04		
		GO:0045893~positive regulation of transcription, DNA-dependent	2.10E-04		
		GO:0042744~hydrogen peroxide catabolic process	2.20E-04		
		GO:0051254~positive regulation of RNA metabolic process	2.20E-04		
		GO:0032501~multicellular organismal process	2.40E-04		
		GO:0070301~cellular response to hydrogen peroxide	2.40E-04		
		GO:0040012~regulation of locomotion	2.40E-04		
		GO:0051270~regulation of cell motion	2.60E-04		
		GO:0044255~cellular lipid metabolic process	2.90E-04		
		GO:0043542~endothelial cell migration	3.00E-04		
		GO:0048514~blood vessel morphogenesis	3.30E-04		
		GO:0045596~negative regulation of cell differentiation	3.80E-04		
		GO:0022603~regulation of anatomical structure morphogenesis	4.10E-04		
		GO:0006464~protein	4.20E-04		

		modification process			
		GO:0050771~negative regulation of axonogenesis	4.30E-04		
		GO:0042743~hydrogen peroxide metabolic process	4.40E-04		
		GO:0051050~positive regulation of transport	4.40E-04		
		GO:0050776~regulation of immune response	4.40E-04		
		GO:0045941~positive regulation of transcription	4.60E-04		
		GO:0033554~cellular response to stress	4.60E-04		
		GO:0045597~positive regulation of cell differentiation	4.60E-04		
		GO:0031345~negative regulation of cell projection organization	4.70E-04		
		GO:0006749~glutathione metabolic process	4.80E-04		
		GO:0010628~positive regulation of gene expression	5.00E-04		
		GO:0002684~positive regulation of immune system process	5.30E-04		
		GO:0009987~cellular process	5.50E-04		
		GO:0034614~cellular response to reactive oxygen species	5.80E-04		
		GO:0043412~biopolymer modification	5.90E-04		
		GO:0001568~blood vessel development	6.10E-04		
		GO:0010646~regulation of cell communication	7.30E-04		
		GO:0022008~neurogenesis	7.40E-04		
		GO:0060341~regulation of cellular localization	8.80E-04		
		GO:0010627~regulation of protein kinase cascade	8.90E-04		
		GO:0001944~vasculature development	9.70E-04		
		GO:0021954~central nervous system neuron development	9.70E-04		
		GO:0031175~neuron projection development	9.90E-04		
		GO:0051093~negative regulation of developmental	1.10E-03		

		process			
		GO:0045935~positive regulation of nucleobase, nucleoside, nucleotide and nucleic acid metabolic process	1.10E-03		
		GO:0031016~pancreas development	1.10E-03		
		GO:0051173~positive regulation of nitrogen compound metabolic process	1.10E-03		
		GO:0048468~cell development	1.20E-03		
		GO:0051094~positive regulation of developmental process	1.20E-03		
		GO:0010557~positive regulation of macromolecule biosynthetic process	1.30E-03		
		GO:0042102~positive regulation of T cell proliferation	1.30E-03		
$M_{(a5)}$	actin, itgb3, klf4, tnfrsf10b, csf3r, csf3, eef1b3, ddit3, nfe2l1, etv4, tbx5, tbx6, tnfsf13, hla-e, myf5, tnnt2, krt8, kdr, nkx2-5, krt7, pax7, tgfb, krt19, plau, ncam1, ervk2, cdkn2a, msmb, apba1, ptgs2, mlh1, dapk1, apba2, brca1, xiap, rassf1, cd40, f2r, src, cftr, bax, ccl18, abcb1, egfr, gata1, cux1, il17ra, mark2, cd14, cyp24a1, pou2f1, slc22a3, slc1a1, plat, tap2, thbd,	GO:0050896~response to stimulus	1.90E-15	Cytokine-cytokine receptor interaction	6.40E-06
		GO:0019882~antigen processing and presentation	1.40E-13	Pathways in cancer	7.10E-05
		GO:0002376~immune system process	1.50E-12	Bladder cancer	1.10E-04
		GO:0042221~response to chemical stimulus	4.10E-12	Antigen processing and presentation	3.60E-04
		GO:0006955~immune response	4.80E-12	Hematopoietic cell lineage	4.30E-04
		GO:0009605~response to external stimulus	7.40E-12	Complement and coagulation cascades	1.10E-03
		GO:0006950~response to stress	1.10E-11	ABC transporters	1.60E-03
		GO:0042981~regulation of apoptosis	4.20E-09	Natural killer cell mediated cytotoxicity	4.10E-03
		GO:0043067~regulation of programmed cell death	5.00E-09	Allograft rejection	8.10E-03
		GO:0009611~response to wounding	5.10E-09	Pancreatic cancer	9.40E-03
		GO:0010941~regulation of cell death	5.40E-09	Cell adhesion molecules (CAMs)	1.80E-02
		GO:0032502~developmental process	7.00E-09		
		GO:0048518~positive regulation of biological process	8.90E-09		

	procr, smad3, bcl2l1, cd1b, krt16, mki67, ccl22, cd1c, gata3, ccl26, hla-b, psmb9, b2m, ccl17, ppa1, tap1, psmb8, nos2, ulbp3, ulbp2, diablo, plaur, cd34, col9a1, ifng, tapbp, th1l, nanog, pou5f1, nfat5, abcb6, f2, cldn7, atp8a2, tymp, stat1, il8, mmp7, hspa5	GO:0009653~anatomical structure morphogenesis	1.50E-08		
		GO:0042127~regulation of cell proliferation	1.60E-08		
		GO:0048856~anatomical structure development	1.80E-08		
		GO:0043281~regulation of caspase activity	3.60E-08		
		GO:0051704~multi-organism process	4.30E-08		
		GO:0006919~activation of caspase activity	4.70E-08		
		GO:0052548~regulation of endopeptidase activity	4.80E-08		
		GO:0007275~multicellular organismal development	5.50E-08		
		GO:0052547~regulation of peptidase activity	7.00E-08		
		GO:0043065~positive regulation of apoptosis	8.00E-08		
		GO:0043068~positive regulation of programmed cell death	8.80E-08		
		GO:0010952~positive regulation of peptidase activity	8.80E-08		
		GO:0043280~positive regulation of caspase activity	8.80E-08		
		GO:0010942~positive regulation of cell death	9.30E-08		
		GO:0009607~response to biotic stimulus	1.30E-07		
		GO:0048513~organ development	1.60E-07		
		GO:0051345~positive regulation of hydrolase activity	2.00E-07		
		GO:0050817~coagulation	2.70E-07		
		GO:0007596~blood coagulation	2.70E-07		
		GO:0006952~defense response	2.80E-07		
		GO:0042060~wound healing	3.60E-07		
		GO:0007599~hemostasis	4.20E-07		
		GO:0040011~locomotion	5.40E-07		
		GO:0048522~positive regulation of cellular process	6.00E-07		
		GO:0048731~system development	9.60E-07		
		GO:0065007~biological	9.70E-07		

		regulation			
		GO:0048583~regulation of response to stimulus	1.30E-06		
		GO:0032501~multicellular organismal process	1.50E-06		
		GO:0051707~response to other organism	2.20E-06		
		GO:0010033~response to organic substance	2.50E-06		
		GO:0050878~regulation of body fluid levels	3.20E-06		
		GO:0002474~antigen processing and presentation of peptide antigen via MHC class I	3.50E-06		
		GO:0007260~tyrosine phosphorylation of STAT protein	4.90E-06		
		GO:0006915~apoptosis	5.60E-06		
		GO:0012501~programmed cell death	6.60E-06		
		GO:0006935~chemotaxis	8.10E-06		
		GO:0042330~taxis	8.10E-06		
		GO:0007626~locomotory behavior	9.40E-06		
		GO:0051336~regulation of hydrolase activity	9.40E-06		
		GO:0044419~interspecies interaction between organisms	1.20E-05		
		GO:0009790~embryonic development	1.30E-05		
		GO:0048646~anatomical structure formation involved in morphogenesis	1.40E-05		
		GO:0002237~response to molecule of bacterial origin	1.80E-05		
		GO:0009888~tissue development	1.80E-05		
		GO:0051240~positive regulation of multicellular organismal process	2.50E-05		
		GO:0048002~antigen processing and presentation of peptide antigen	2.80E-05		
		GO:0009617~response to bacterium	3.10E-05		
		GO:0031325~positive regulation of cellular	3.50E-05		

		metabolic process			
		GO:0006917~induction of apoptosis	3.60E-05		
		GO:0012502~induction of programmed cell death	3.70E-05		
		GO:0048519~negative regulation of biological process	4.10E-05		
		GO:0008219~cell death	4.50E-05		
		GO:0006928~cell motion	4.60E-05		
		GO:0050789~regulation of biological process	4.70E-05		
		GO:0016265~death	4.90E-05		
		GO:0051173~positive regulation of nitrogen compound metabolic process	5.30E-05		
		GO:0048514~blood vessel morphogenesis	5.90E-05		
		GO:0009893~positive regulation of metabolic process	6.20E-05		
		GO:0016477~cell migration	6.50E-05		
		GO:0048523~negative regulation of cellular process	8.00E-05		
		GO:0044093~positive regulation of molecular function	8.30E-05		
		GO:0001775~cell activation	8.80E-05		
		GO:0010604~positive regulation of macromolecule metabolic process	9.20E-05		
		GO:0043085~positive regulation of catalytic activity	1.10E-04		
		GO:0032879~regulation of localization	1.20E-04		
		GO:0051674~localization of cell	1.50E-04		
		GO:0048870~cell motility	1.50E-04		
		GO:0045935~positive regulation of nucleobase, nucleoside, nucleotide and nucleic acid metabolic process	1.60E-04		
		GO:0001568~blood vessel development	1.70E-04		
		GO:0030154~cell differentiation	1.80E-04		
		GO:0007610~behavior	1.90E-04		
		GO:0001944~vasculature	2.00E-04		

		development			
		GO:0018108~peptidyl-tyrosine phosphorylation	2.10E-04		
		GO:0048660~regulation of smooth muscle cell proliferation	2.10E-04		
		GO:0006954~inflammatory response	2.20E-04		
		GO:0009987~cellular process	2.20E-04		
		GO:0022402~cell cycle process	2.40E-04		
		GO:0060255~regulation of macromolecule metabolic process	2.40E-04		
		GO:0018212~peptidyl-tyrosine modification	2.40E-04		
		GO:0051239~regulation of multicellular organismal process	2.60E-04		
		GO:0065008~regulation of biological quality	2.90E-04		
		GO:0008284~positive regulation of cell proliferation	3.00E-04		
		GO:0048869~cellular developmental process	3.30E-04		
		GO:0002706~regulation of lymphocyte mediated immunity	3.80E-04		
		GO:0002822~regulation of adaptive immune response based on somatic recombination of immune receptors built from immunoglobulin superfamily domains	4.10E-04		
		GO:0043066~negative regulation of apoptosis	4.20E-04		
		GO:0019222~regulation of metabolic process	4.30E-04		
		GO:0002819~regulation of adaptive immune response	4.40E-04		
		GO:0051049~regulation of transport	4.40E-04		
		GO:0009891~positive regulation of biosynthetic process	4.40E-04		
		GO:0031343~positive regulation of cell killing	4.60E-04		

		GO:0002697~regulation of immune effector process	4.60E-04		
		GO:0043069~negative regulation of programmed cell death	4.60E-04		
		GO:0060548~negative regulation of cell death	4.70E-04		
		GO:0008285~negative regulation of cell proliferation	4.80E-04		
		GO:0007050~cell cycle arrest	5.00E-04		
		GO:0032101~regulation of response to external stimulus	5.30E-04		
		GO:0043627~response to estrogen stimulus	5.50E-04		
		GO:0007243~protein kinase cascade	5.80E-04		
		GO:0000018~regulation of DNA recombination	5.90E-04		
		GO:0002703~regulation of leukocyte mediated immunity	6.10E-04		
		GO:0031341~regulation of cell killing	7.30E-04		
		GO:0010605~negative regulation of macromolecule metabolic process	7.40E-04		
		GO:0010557~positive regulation of macromolecule biosynthetic process	8.80E-04		
		GO:0045941~positive regulation of transcription	8.90E-04		
		GO:0014706~striated muscle tissue development	9.70E-04		
		GO:0051924~regulation of calcium ion transport	9.70E-04		
		GO:0008637~apoptotic mitochondrial changes	9.90E-04		
		GO:0009719~response to endogenous stimulus	1.10E-03		
		GO:0040007~growth	1.10E-03		
		GO:0065009~regulation of molecular function	1.10E-03		
		GO:0010628~positive regulation of gene expression	1.10E-03		
		GO:0051928~positive regulation of calcium ion transport	1.20E-03		
		GO:0060537~muscle tissue development	1.20E-03		

		GO:0009892~negative regulation of metabolic process	1.30E-03		
		GO:0031328~positive regulation of cellular biosynthetic process	1.30E-03		
		GO:0045830~positive regulation of isotype switching	1.40E-03		
		GO:0045911~positive regulation of DNA recombination	1.40E-03		
		GO:0048545~response to steroid hormone stimulus	1.40E-03		
		GO:0040012~regulation of locomotion	1.40E-03		
		GO:0050794~regulation of cellular process	1.40E-03		
		GO:0032496~response to lipopolysaccharide	1.50E-03		
		GO:0031667~response to nutrient levels	1.60E-03		
		GO:0043403~skeletal muscle regeneration	1.70E-03		
		GO:0010959~regulation of metal ion transport	1.80E-03		
		GO:0007259~JAK-STAT cascade	1.90E-03		
		GO:0031324~negative regulation of cellular metabolic process	2.00E-03		
		GO:0006916~anti-apoptosis	2.00E-03		
		GO:0002478~antigen processing and presentation of exogenous peptide antigen	2.10E-03		
		GO:0009725~response to hormone stimulus	2.30E-03		
		GO:0051146~striated muscle cell differentiation	2.40E-03		
		GO:0043270~positive regulation of ion transport	2.40E-03		
		GO:0051246~regulation of protein metabolic process	2.50E-03		
		GO:0050790~regulation of catalytic activity	2.60E-03		
		GO:0031399~regulation of protein modification process	2.80E-03		
		GO:0009991~response to extracellular stimulus	2.80E-03		

		GO:0080090~regulation of primary metabolic process	2.90E-03		
		GO:0045191~regulation of isotype switching	3.00E-03		
		GO:0009887~organ morphogenesis	3.10E-03		
		GO:0032268~regulation of cellular protein metabolic process	3.20E-03		
		GO:0050776~regulation of immune response	3.20E-03		
		GO:0006355~regulation of transcription, DNA-dependent	3.30E-03		
		GO:0045893~positive regulation of transcription, DNA-dependent	3.30E-03		
		GO:0019884~antigen processing and presentation of exogenous antigen	3.50E-03		
		GO:0031323~regulation of cellular metabolic process	3.50E-03		
		GO:0051254~positive regulation of RNA metabolic process	3.50E-03		
		GO:0040017~positive regulation of locomotion	3.50E-03		
		GO:0044057~regulation of system process	3.60E-03		
		GO:0050793~regulation of developmental process	3.60E-03		
		GO:0043269~regulation of ion transport	3.70E-03		
		GO:0007049~cell cycle	3.70E-03		
		GO:0048584~positive regulation of response to stimulus	4.00E-03		
		GO:0050864~regulation of B cell activation	4.20E-03		
		GO:0051252~regulation of RNA metabolic process	4.30E-03		
		GO:0045321~leukocyte activation	4.50E-03		
		GO:0007154~cell communication	4.50E-03		
		GO:0030334~regulation of cell migration	4.50E-03		
		GO:0007267~cell-cell signaling	4.80E-03		

		GO:0008629~induction of apoptosis by intracellular signals	4.90E-03		
		GO:0060541~respiratory system development	5.00E-03		
		GO:0051179~localization	5.00E-03		
		GO:0010647~positive regulation of cell communication	5.00E-03		
		GO:0055001~muscle cell development	5.40E-03		
		GO:0051054~positive regulation of DNA metabolic process	5.40E-03		
		GO:0048729~tissue morphogenesis	5.90E-03		
		GO:0051052~regulation of DNA metabolic process	6.10E-03		
		GO:0050927~positive regulation of positive chemotaxis	6.40E-03		
		GO:0050926~regulation of positive chemotaxis	6.40E-03		
		GO:0032651~regulation of interleukin-1 beta production	7.10E-03		
		GO:0042692~muscle cell differentiation	7.40E-03		
		GO:0001894~tissue homeostasis	7.60E-03		
		GO:0048468~cell development	7.80E-03		
		GO:0001912~positive regulation of leukocyte mediated cytotoxicity	7.80E-03		
		GO:0045884~regulation of survival gene product expression	7.80E-03		
		GO:0001836~release of cytochrome c from mitochondria	7.80E-03		
		GO:0051270~regulation of cell motion	7.80E-03		
		GO:0046649~lymphocyte activation	8.80E-03		
		GO:0042698~ovulation cycle	8.90E-03		
		GO:0032652~regulation of interleukin-1 production	9.30E-03		
		GO:0010035~response to	1.00E-02		

		inorganic substance			
		GO:0002712~regulation of B cell mediated immunity	1.00E-02		
		GO:0002889~regulation of immunoglobulin mediated immune response	1.00E-02		
		GO:0050678~regulation of epithelial cell proliferation	1.00E-02		
		GO:0001666~response to hypoxia	1.10E-02		
		GO:0001910~regulation of leukocyte mediated cytotoxicity	1.10E-02		
		GO:0007517~muscle organ development	1.10E-02		
		GO:0030155~regulation of cell adhesion	1.10E-02		
		GO:0002682~regulation of immune system process	1.10E-02		
		GO:0007005~mitochondrion organization	1.20E-02		
		GO:0002637~regulation of immunoglobulin production	1.20E-02		
		GO:0045165~cell fate commitment	1.20E-02		
		GO:0007584~response to nutrient	1.20E-02		
		GO:0070482~response to oxygen levels	1.30E-02		
		GO:0002477~antigen processing and presentation of exogenous peptide antigen via MHC class Ib	1.30E-02		
		GO:0002428~antigen processing and presentation of peptide antigen via MHC class Ib	1.30E-02		
		GO:0002481~antigen processing and presentation of exogenous protein antigen via MHC class Ib, TAP-dependent	1.30E-02		
		GO:0001952~regulation of cell-matrix adhesion	1.30E-02		
		GO:0051050~positive regulation of transport	1.40E-02		
		GO:0034097~response to cytokine stimulus	1.40E-02		
		GO:0050921~positive	1.50E-02		

		regulation of chemotaxis			
		GO:0001525~angiogenesis	1.50E-02		
		GO:0010629~negative regulation of gene expression	1.50E-02		
		GO:0006461~protein complex assembly	1.50E-02		
		GO:0070271~protein complex biogenesis	1.50E-02		
		GO:0007423~sensory organ development	1.60E-02		
		GO:0001501~skeletal system development	1.60E-02		
		GO:0031644~regulation of neurological system process	1.70E-02		
		GO:0048661~positive regulation of smooth muscle cell proliferation	1.70E-02		
		GO:0050920~regulation of chemotaxis	1.70E-02		
		GO:0048871~multicellular organismal homeostasis	1.70E-02		
		GO:0030168~platelet activation	1.80E-02		
		GO:0042246~tissue regeneration	1.80E-02		
		GO:0010468~regulation of gene expression	1.80E-02		
		GO:0002684~positive regulation of immune system process	1.80E-02		
		GO:0048589~developmental growth	1.80E-02		
		GO:0050871~positive regulation of B cell activation	1.90E-02		
		GO:0051712~positive regulation of killing of cells of another organism	1.90E-02		
		GO:0043902~positive regulation of multi-organism process	1.90E-02		
		GO:0014909~smooth muscle cell migration	1.90E-02		
		GO:0043009~chordate embryonic development	1.90E-02		
		GO:0030335~positive regulation of cell migration	1.90E-02		
		GO:0000060~protein import into nucleus, translocation	2.00E-02		

		GO:0034976~response to endoplasmic reticulum stress	2.00E-02		
		GO:0048520~positive regulation of behavior	2.00E-02		
		GO:0001819~positive regulation of cytokine production	2.00E-02		
		GO:0009792~embryonic development ending in birth or egg hatching	2.00E-02		
		GO:0018193~peptidyl-amino acid modification	2.00E-02		
		GO:0006979~response to oxidative stress	2.10E-02		
		GO:0051241~negative regulation of multicellular organismal process	2.10E-02		
		GO:0006984~ER-nuclear signaling pathway	2.10E-02		
		GO:0008283~cell proliferation	2.10E-02		
		GO:0016043~cellular component organization	2.20E-02		
		GO:0001707~mesoderm formation	2.20E-02		
		GO:0030193~regulation of blood coagulation	2.20E-02		
		GO:0046907~intracellular transport	2.40E-02		
		GO:0048332~mesoderm morphogenesis	2.40E-02		
		GO:0051272~positive regulation of cell motion	2.50E-02		
		GO:0044085~cellular component biogenesis	2.50E-02		
		GO:0022607~cellular component assembly	2.50E-02		
		GO:0051890~regulation of cardioblast differentiation	2.50E-02		
		GO:0051891~positive regulation of cardioblast differentiation	2.50E-02		
		GO:0007262~STAT protein nuclear translocation	2.50E-02		
		GO:0051709~regulation of killing of cells of another organism	2.50E-02		
		GO:0030324~lung development	2.50E-02		

		GO:0031396~regulation of protein ubiquitination	2.60E-02		
		GO:0001704~formation of primary germ layer	2.70E-02		
		GO:0007242~intracellular signaling cascade	2.70E-02		
		GO:0030323~respiratory tube development	2.70E-02		
		GO:0032269~negative regulation of cellular protein metabolic process	2.80E-02		
		GO:0002700~regulation of production of molecular mediator of immune response	2.80E-02		
		GO:0001541~ovarian follicle development	2.80E-02		
		GO:0050818~regulation of coagulation	2.80E-02		
		GO:0001817~regulation of cytokine production	2.80E-02		
		GO:0051171~regulation of nitrogen compound metabolic process	2.90E-02		
		GO:0060249~anatomical structure homeostasis	3.00E-02		
		GO:0045944~positive regulation of transcription from RNA polymerase II promoter	3.10E-02		
		GO:0031077~post-embryonic camera-type eye development	3.10E-02		
		GO:0002475~antigen processing and presentation via MHC class Ib	3.10E-02		
		GO:0014812~muscle cell migration	3.10E-02		
		GO:0016337~cell-cell adhesion	3.20E-02		
		GO:0031401~positive regulation of protein modification process	3.20E-02		
		GO:0051248~negative regulation of protein metabolic process	3.20E-02		
		GO:0002699~positive regulation of immune effector process	3.20E-02		
		GO:0051641~cellular	3.40E-02		

		localization			
		GO:0007155~cell adhesion	3.40E-02		
		GO:0022610~biological adhesion	3.40E-02		
		GO:0010810~regulation of cell-substrate adhesion	3.50E-02		
		GO:0050795~regulation of behavior	3.50E-02		
		GO:0008624~induction of apoptosis by extracellular signals	3.50E-02		
		GO:0051174~regulation of phosphorus metabolic process	3.50E-02		
		GO:0019220~regulation of phosphate metabolic process	3.50E-02		
		GO:0051716~cellular response to stimulus	3.60E-02		
		GO:0032844~regulation of homeostatic process	3.60E-02		
		GO:0001503~ossification	3.70E-02		
		GO:0045595~regulation of cell differentiation	3.70E-02		
		GO:0032735~positive regulation of interleukin-12 production	3.80E-02		
		GO:0048304~positive regulation of isotype switching to IgG isotypes	3.80E-02		
		GO:0019885~antigen processing and presentation of endogenous peptide antigen via MHC class I	3.80E-02		
		GO:0002483~antigen processing and presentation of endogenous peptide antigen	3.80E-02		
		GO:0006351~transcription, DNA-dependent	3.90E-02		
		GO:0009314~response to radiation	3.90E-02		
		GO:0009967~positive regulation of signal transduction	4.00E-02		
		GO:0016202~regulation of striated muscle tissue development	4.00E-02		
		GO:0031400~negative regulation of protein modification process	4.00E-02		

		GO:0032774~RNA biosynthetic process	4.10E-02		
		GO:0006357~regulation of transcription from RNA polymerase II promoter	4.20E-02		
		GO:0043524~negative regulation of neuron apoptosis	4.20E-02		
		GO:0048634~regulation of muscle development	4.20E-02		
		GO:0055002~striated muscle cell development	4.30E-02		
		GO:0010922~positive regulation of phosphatase activity	4.40E-02		
		GO:0001916~positive regulation of T cell mediated cytotoxicity	4.40E-02		
		GO:0008634~negative regulation of survival gene product expression	4.40E-02		
		GO:0051968~positive regulation of synaptic transmission, glutamatergic	4.40E-02		
		GO:0060348~bone development	4.40E-02		
		GO:0051649~establishment of localization in cell	4.40E-02		
		GO:0045934~negative regulation of nucleobase, nucleoside, nucleotide and nucleic acid metabolic process	4.50E-02		
		GO:0019219~regulation of nucleobase, nucleoside, nucleotide and nucleic acid metabolic process	4.50E-02		
		GO:0048598~embryonic morphogenesis	4.60E-02		
		GO:0009889~regulation of biosynthetic process	4.70E-02		
		GO:0034613~cellular protein localization	4.70E-02		
		GO:0051172~negative regulation of nitrogen compound metabolic process	4.80E-02		
		GO:0010556~regulation of macromolecule biosynthetic process	4.80E-02		
		GO:0048511~rhythmic	4.80E-02		

		process			
		GO:0070727~cellular macromolecule localization	4.90E-02		
		GO:0042493~response to drug	4.90E-02		
		GO:0010038~response to metal ion	4.90E-02		
$M_{(a6)}$	ugt1a9, ugt1a6, ugt2b15, slc35a2, ugt2b7, ugt1a4, ugt1a1, ugt1a7, ugt1a3, ugt1a10	GO:0006805~xenobiotic metabolic process	4.20E-03	Ascorbate and aldarate metabolism	1.10E-05
		GO:0009410~response to xenobiotic stimulus	4.90E-03	Pentose and glucuronate interconversions	1.20E-05
		GO:0006629~lipid metabolic process	9.60E-03	Porphyrin and chlorophyll metabolism	4.10E-05
		GO:0008202~steroid metabolic process	4.20E-02	Androgen and estrogen metabolism	5.20E-05
				Starch and sucrose metabolism	6.70E-05
				Drug metabolism	7.00E-05
				Steroid hormone biosynthesis	8.00E-05
				Retinol metabolism	1.10E-04
				Metabolism of xenobiotics by cytochrome P450	1.40E-04
				Drug metabolism	1.50E-04
$M_{(a7)}$	apoe, apoa2, apoa1, nqo1, apob, apoa4, selp, ttr, sod2, nfe2l2, apoc3, gabpa, apoh, ros1, hmox1, scarb1, apom, s1pr2, s1pr3, pah, a2m, il1b, hsd11b1, mbtps1, s1pr1, abca1	-		PPAR signaling pathway	1.50E-02
$M_{(a8)}$	vhl, iqsec1, lrrn1, foxp1, fgd5, gnai2, bcl6, aldh1l1, ctdspl	GO:0042127~regulation of cell proliferation	3.00E-03	-	
		GO:0046578~regulation of Ras protein signal transduction	3.20E-03		
		GO:0051056~regulation of small GTPase mediated signal transduction	4.50E-03		
		GO:0000122~negative regulation of transcription from RNA polymerase II promoter	5.00E-03		
		GO:0010646~regulation of cell communication	6.70E-03		
		GO:0045892~negative	8.90E-03		

		regulation of transcription, DNA-dependent			
		GO:0051253~negative regulation of RNA metabolic process	9.20E-03		
		GO:0002637~regulation of immunoglobulin production	1.10E-02		
		GO:0032319~regulation of Rho GTPase activity	1.20E-02		
		GO:0008284~positive regulation of cell proliferation	1.20E-02		
		GO:0016481~negative regulation of transcription	1.50E-02		
		GO:0002700~regulation of production of molecular mediator of immune response	1.70E-02		
		GO:0010629~negative regulation of gene expression	1.70E-02		
		GO:0045934~negative regulation of nucleobase, nucleoside, nucleotide and nucleic acid metabolic process	1.80E-02		
		GO:0051172~negative regulation of nitrogen compound metabolic process	1.80E-02		
		GO:0030183~B cell differentiation	2.00E-02		
		GO:0010558~negative regulation of macromolecule biosynthetic process	2.00E-02		
		GO:0031327~negative regulation of cellular biosynthetic process	2.10E-02		
		GO:0009890~negative regulation of biosynthetic process	2.20E-02		
		GO:0048523~negative regulation of cellular process	2.50E-02		
		GO:0050793~regulation of developmental process	3.00E-02		
		GO:0048519~negative regulation of biological process	3.10E-02		
		GO:0042113~B cell activation	3.20E-02		
		GO:0048522~positive regulation of cellular process	3.30E-02		
		GO:0031324~negative regulation of cellular	3.40E-02		

		metabolic process			
		GO:0006357~regulation of transcription from RNA polymerase II promoter	3.50E-02		
		GO:0010605~negative regulation of macromolecule metabolic process	3.50E-02		
		GO:0009892~negative regulation of metabolic process	3.90E-02		
		GO:0035023~regulation of Rho protein signal transduction	4.10E-02		
		GO:0002697~regulation of immune effector process	4.20E-02		
		GO:0048518~positive regulation of biological process	4.20E-02		
		GO:0030098~lymphocyte differentiation	4.30E-02		
		GO:0032318~regulation of Ras GTPase activity	4.30E-02		
		GO:0050790~regulation of catalytic activity	4.60E-02		
		GO:0009966~regulation of signal transduction	4.90E-02		
$M_{(a9)}$	sco1, slc39a8, fcgr3b, c3ar1, retn, rad21, slc3a2, lrrc25	GO:0030001~metal ion transport	1.50E-02	-	
		GO:0006812~cation transport	2.10E-02		
		GO:0000041~transition metal ion transport	3.00E-02		
		GO:0006811~ion transport	3.80E-02		
$M_{(a10)}$	flt1, phactr1, cbs, lpl, psrc1, cnnm2, cdkn2b	GO:0048522~positive regulation of cellular process	1.30E-03	-	
		GO:0048518~positive regulation of biological process	2.00E-03		
		GO:0050794~regulation of cellular process	9.50E-03		
		GO:0009966~regulation of signal transduction	1.10E-02		
		GO:0050789~regulation of biological process	1.20E-02		
		GO:0010646~regulation of cell communication	1.30E-02		
		GO:0065007~biological regulation	1.60E-02		

		GO:0016053~organic acid biosynthetic process	4.00E-02		
		GO:0046394~carboxylic acid biosynthetic process	4.00E-02		
		GO:0045859~regulation of protein kinase activity	4.60E-02		
		GO:0043549~regulation of kinase activity	4.90E-02		
